# 3D MALDI mass spectrometry imaging reveals specific localization of long-chain acylcarnitines within a 10-day time window of spinal cord injury

**DOI:** 10.1038/s41598-018-34518-0

**Published:** 2018-10-31

**Authors:** Jusal Quanico, Lena Hauberg-Lotte, Stephanie Devaux, Zahra Laouby, Celine Meriaux, Antonella Raffo-Romero, Melanie Rose, Leia Westerheide, Jost Vehmeyer, Franck Rodet, Peter Maass, Dasa Cizkova, Norbert Zilka, Veronika Cubinkova, Isabelle Fournier, Michel Salzet

**Affiliations:** 1 Université de Lille, INSERM U1192 - Laboratoire Protéomique, Réponse Inflammatoire et Spectrométrie de Masse (PRISM), F-59000 Lille, France; 2 Center for Industrial Mathematics, University of Bremen, Bibliothekstraße 5, 28359 Bremen, Germany; 3 Department of Anatomy, Histology and Physiology, University of Veterinary Medicine and Pharmacy in Kosice, Komenskeho 73, 041 81 Kosice, Slovakia; 4 Institute of Neuroimmunology, Slovak Academy of Sciences, Dubravska cesta 9, 845 10 Bratislava, Slovakia

## Abstract

We report, for the first time, the detection and specific localization of long-chain acylcarnitines (LC ACs) along the lesion margins in an experimental model of spinal cord injury (SCI) using 3D mass spectrometry imaging (MSI). Acylcarnitines palmitoylcarnitine (AC(16:0)), palmitoleoylcarnitine (AC(16:1)), elaidic carnitine (AC(18:1)) and tetradecanoylcarnitine (AC(14:1)) were detected as early as 3 days post injury, and were present along the lesion margins 7 and 10 days after SCI induced by balloon compression technique in the rat. 3D MSI revealed the heterogeneous distribution of these lipids across the injured spinal cord, appearing well-defined at the lesion margins rostral to the lesion center, and becoming widespread and less confined to the margins at the region located caudally. The assigned acylcarnitines co-localize with resident microglia/macrophages detected along the lesion margins by immunofluorescence. Given the reported pro-inflammatory role of these acylcarnitines, their specific spatial localization along the lesion margin could hint at their potential pathophysiological roles in the progression of SCI.

## Introduction

SCI is a devastating medical condition leading to irreversible damage of the central nervous system (CNS). Traumatic SCI can lead to paralysis with complete or partial loss of neurological functions below the injury site, and this can result from several different causes such as road traffic crashes, falls, and violence^1^. Nowadays, the increased incidence of trauma may be related to popular sports such as ice hockey, American football, rugby, horse riding and diving^2,3^. Currently, there are no effective therapies available for SCI patients. The long-standing challenge facing researchers is to develop effective strategies to prevent further tissue loss, maintain the health of living cells, and replace cells that have died to enable axonal growth and reestablish synapses that restore neural circuits essential for proper functional recovery^4^.

A key factor for effective therapy is elucidation of the distinct phases involved in SCI and the cellular and molecular events underlying them^3^. Diverse groups of cells and molecules from the nervous, immune, and vascular systems are implicated. Most participating cells reside in the spinal cord; however, others are translocated to the site of injury from the circulatory system. Thus, after primary trauma, cellular and molecular injury and inflammatory cascades are initiated, causing activation of resident microglia and astrocytes coupled with infiltration of innate immune cells including lymphocytes and monocytes. Furthermore, the local release of cytokines and chemokines by microglia, macrophages and neural cells induces a particular environment that can be either neurotoxic or neurotrophic^4–6^. During acute phase, macrophages phagocyte cell debris and glial scar formation is hypothesized to protect healthy tissue^7^. Chronic inflammatory processes (weeks post trauma) lead to aberrant tissue remodeling and nerve tissue dysfunction. Various cellular and molecular events designed to heal the injury can paradoxically lead to further neuronal injury or even cell death. The site of injury may spread to adjacent areas of the spinal cord, sometimes extending four spinal segments above and below the initial lesion site. The affected area markedly expands, becomes filled with immune cells, and a “scar” is formed^7^.

One of the approved clinical treatments for SCI is administration of methylprednisolone that can modulate the inflammatory process. However, a high-dose of methylprednisolone is often associated with severe immunosuppression and side effects, such as pulmonary or urinary tract infections^8,9^. In addition to mono-therapies, more complex cellular therapies are being suggested carrying several advantages and targeting several SCI-associated conditions such as: to bridge cavities or cysts, to replace dead cells, to create a favorable environment, and to allow axonal regeneration^8–10^. However, none of these provides a total understanding of the injury-inflammatory mechanisms involved in the lesioned spinal cord and proximities that can be used for a temporal and segment-specific target in SCI treatment. Thus, the molecular cross-talk occurring among cellular inhabitants at the lesion site and the adjacent segments needs to be investigated for this purpose.

Thus, in order to get an accurate view of the injury-driven mechanisms where the inflammatory process and neural injury are implicated, we have extended our previous analysis^5^ to involve a spatiotemporal lipidomic evaluation by carrying out 3D Matrix-Assisted Laser Desorption/Ionization (MALDI) MS imaging across the SCI tissue. Combined with the most advanced tools for processing and statistical analysis of MSI datasets, we demonstrate the advantage of this molecular imaging method in probing SCI to provide novel insights into its pathophysiological mechanism.

## Results

### 2D MSI reveals lesion-specific lipids after SCI

**Figure 1:**
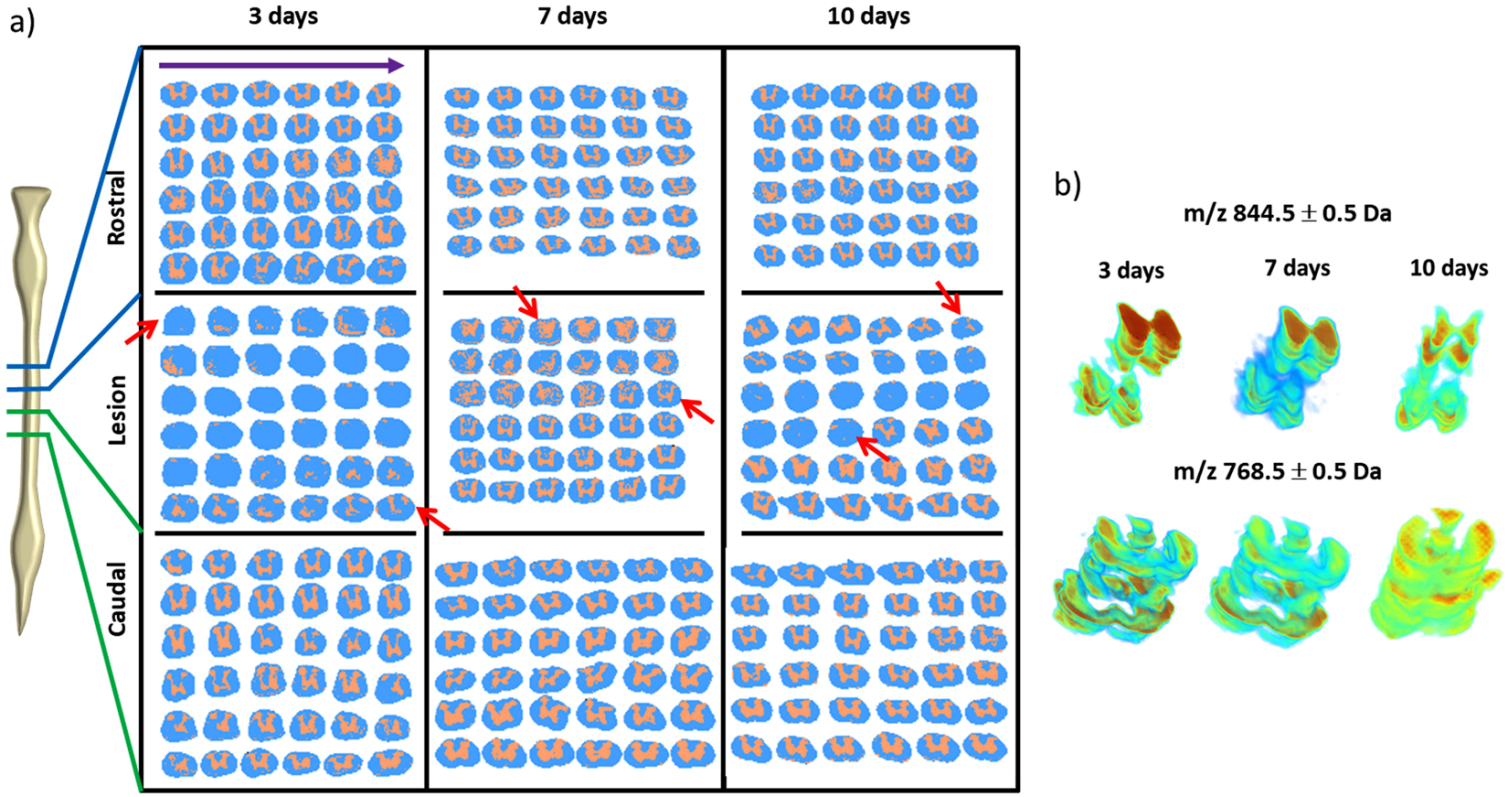
Mapping of spatial segmentation results using all imaged tissue sections. (**a**) Segmentation was obtained by k-means clustering using Euclidean distance as metric. The segmentation maps represent the two main branches comprised of spectra from the gray matter (flesh) and white matter (light blue) regions. Sections in L show significant disruption of molecular content indicated by the loss of the “butterfly” shape typical of the gray matter region (red arrows mark the beginning and end of loss of butterfly shape). The order by which the sections were taken from the spinal cord is from left to right on each row (indicated by the violet arrow). (**b**) Stacking of the dissected ion images of m/z 844.5 and m/z 768.5 representing the gray and white matter regions, respectively. Distortion of ion distribution due to the lesion is more prominent in the gray matter.

2D MALDI MS imaging of uninjured rat spinal cord typically shows distinct distribution of different lipid species. They are contained within the gray and white matter, resulting in spectra clustering according to these two regions, *e.g*., distribution of PC [16:0/16:0], m/z 830.5 and m/z 768.6 in sections taken from the cervical lower C5-C6 (R2) and lumbar L6-S1 segments (C3) that were unaffected by the lesion, where the distinct localization of these signals in the dorsal horn, the rest of the gray matter (intermediate zone and ventral horn), and the white matter, respectively, can be observed (Supplementary Fig. S1). When the pixels are colored based on these clusters, the well-recognizable “butterfly” shape of the gray matter is evident, with the posterior and anterior gray horns well-defined (Fig. 1a). Upon induction of SCI at thoracic segments Th8-Th9, disruption of this distribution is observed at the lesion center and penumbra, leading to the disappearance of high MW signals (ie., m/z 750–1000) and generation of low-MW signals (ie., m/z 300–600) in all areas affected by the lesion, *i.e*., at the lesion site (L) itself as well as in the caudal parts of R1 (adjacent segment rostral to injury) and rostral parts of C1 (adjacent segment caudal to injury). Consequently, the area attributed to the gray matter disappears or appears to be distorted in sections taken close to the lesion center at this and at 7 and 10 days post-SCI (Fig. 1b).

**Figure 2:**
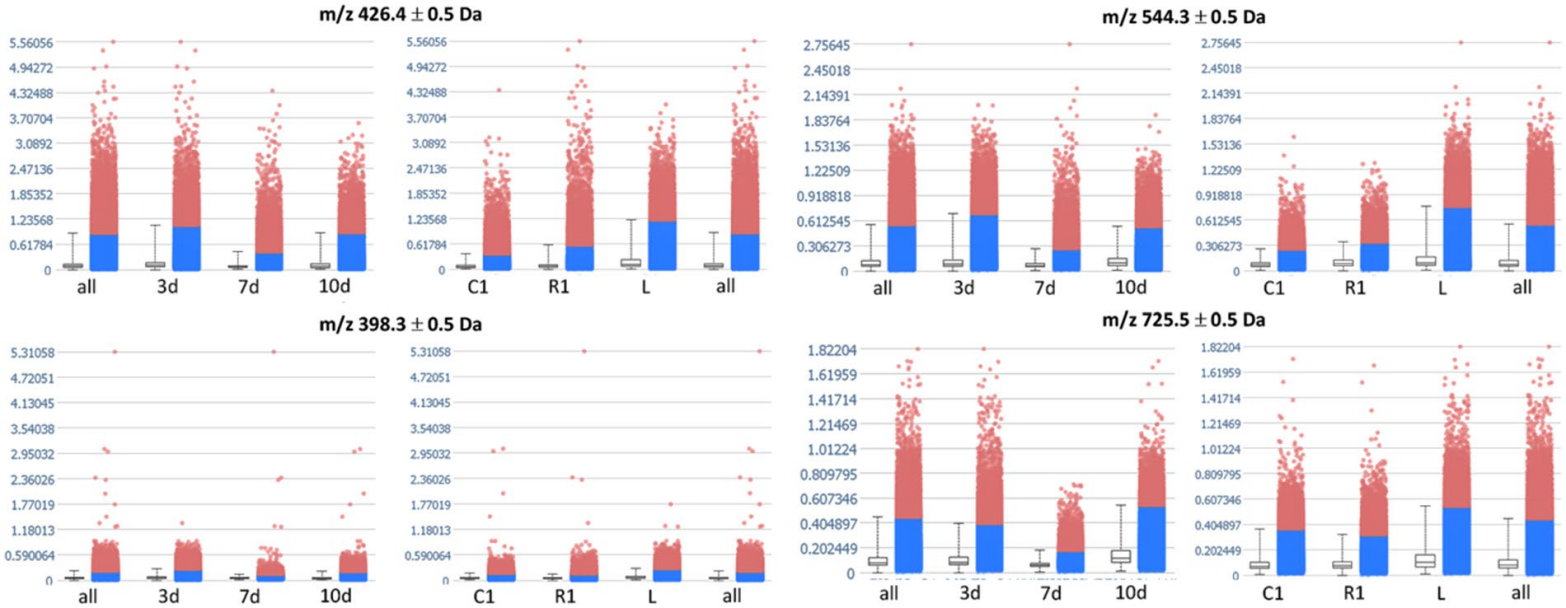
Comparison of the relative intensities of selected lesion-co-localized m/z in all spectra grouped according to time point (left) and segment (right). Majority of the co-localized intervals have elevated intensities at the lesion segment and at 3 days after SCI (long-chain acylcarnitines AC(18:1) (m/z 426.4), AC(16:1) (m/z 398.3) and lysoPC(18:1) (m/z 544.3); a few, such as m/z 725.5, have more intense signals at the lesion segment and at 10 days after SCI. Data points in red correspond to spectra whose intensities at the particular m/z are outside the confidence interval.

Automatic elucidation of co-localized m/z by Pearson’s correlation analysis confirms the lower intensities of high-MW signals at the lesion site (Supplementary Table S1). At the lesion site instead, Pearson’s correlation highlights that predominantly co-localized signals are low-MW. Among these, LC ACs AC(16:1) (m/z 398.3), AC(16:0) (m/z 400.3), and AC(18:1) (m/z 426.4) and an oxidized long-chain fatty acid FA 26:1(9OH,10OH) (m/z 427.4) are specific to this area, in addition to being co-localized at the lesion site. This finding is cross-validated by ROC analysis (Supplementary Table S2). Indeed, pairwise comparison of spectra taken from the lesion (L) segments at each time point post-SCI reveals that these signals are present at all time points. Comparison of the intensities of these m/z, normalized against the total ion count (TIC), and grouped according to the different time points and segments, shows that most of them (AC(16:1), AC(18:1), lysophosphatidylcholines lysoPC(16:0) (m/z 518.3) and lysoPC(18:1) (m/z 544.3), m/z 534.3 and m/z 562.3) have more elevated intensities in the lesion segment and at 3 days post lesion (Fig. 2**)**. On the other hand, few m/z (m/z 721.6, m/z 722.5, and m/z 725.5) also exhibit increased intensities at the lesion segment but are more intense at 10 days after lesion.

Examination of the time point-specific, lesion-discriminative m/z shows that most of the signals associated with the SCI lesion at 3 days are low-MW, while those associated with the lesion at 10 days are high-MW (examples shown in Fig. S2). The latter includes PC [16:0/20:4] (m/z 820.5), previously identified as one of the arachidonic acid-containing PCs and temporarily elevated at the SCI impact site^11^. Other m/z include those observed in normal spinal cord (m/z 770.5, m/z 772.6), as the injured site 10 days post-lesion is less extended most probably due to glial scar formation^4^.

**Figure 3:**
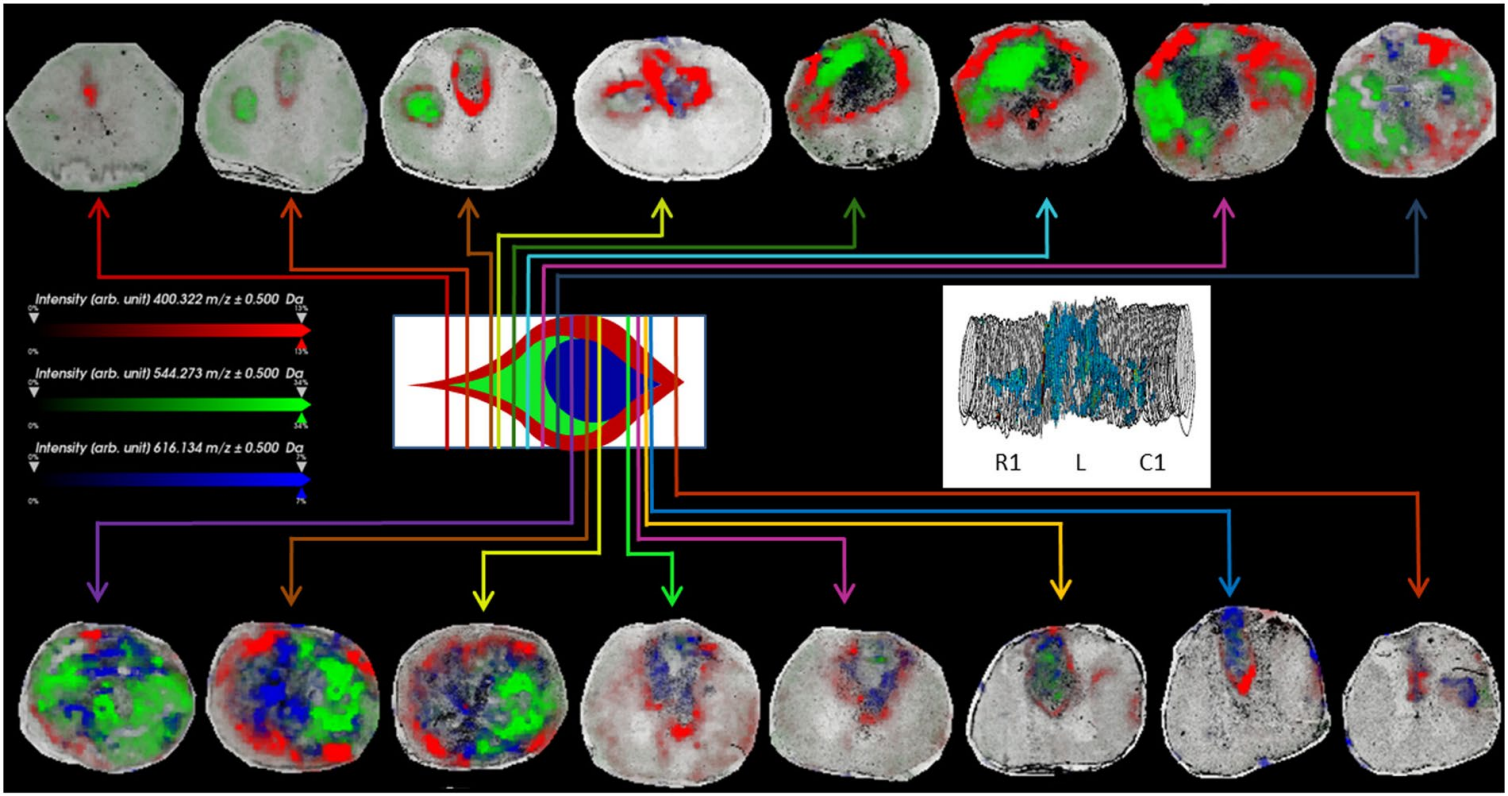
Composite images of AC(16:0) (m/z 400.3, red), lysoPC(18:1) (m/z 544.3, green) and heme fragment of hemoglobin (m/z 616.2, blue) in sections taken at various locations along the rostro-caudal axis of the spinal cord 3 days after injury, as indicated in the idealized cross-sectional diagram of the lesion site. Inset shows the 3D image stack of AC(16:0) in all sections from segments R1, L and C1 subjected to MSI for SCI lesion at 3 days, showing the turnip shape of the lesion.

Detailed examination of the lesion site, by imaging of the lesion-co-localized ions, highlights further sub-structuring within the lesion. As shown in Fig. 3 at 3 days post-SCI, AC(16:0) (as well as AC(16:1) and AC(18:1)) presents a distinct distribution surrounding the lesion site. On the other hand, lysoPC(18:1) (as well as lysoPC(16:0) and lysoPC(18:0) (m/z 546.3), and m/z 534.3) are distinctly distributed within the lesion core not overlapping the surrounding ions. In addition, the heme fragment of hemoglobin (m/z 616.2) is also observed distinct from that of the other ions indicating that the specific distribution of lipids is not related to the hemorrhage. Similar patterns of lipid distribution were found at 7 and 10 days post-injury confirming that this particular distribution is not specific to a single time point (Supplementary Figs S3, S4).

### 3D MSI demonstrates the heterogeneous shape of lesion

The heterogeneity of the distribution of AC(16:0) can be further assessed by generating the 3D projection of the ion images. In the rostral end of the lesion, AC(16:0) ion distribution initially appears as a localized spot observable at the dorsal column, particularly at the site of descending corticospinal tract/pyramidal tract (CST, Fig. 3). In succeeding sections approaching the lesion center, the distribution is expanded and begins to outline affected tissue, eventually covering the remnants of the disrupted gray matter, until it borders the entire girth of the spinal cord at the lesion center. From then on and going towards the caudal end of the lesion, the outline thickens, with the inner diameter increasing as the site of affected tissue recedes. At a certain point along the caudal end of the lesion, the thickening collapses and the ring contracts and reverts to outlining the site of injury, eventually terminating as a spot observable at the dorsal column as was observed in the rostral part. Thus, following AC(16:0) shows a hollow, turnip-shaped distribution across the segment with position-dependent variation in thickness, outlining the site of lesion (Fig. 3).

**Figure 4:**
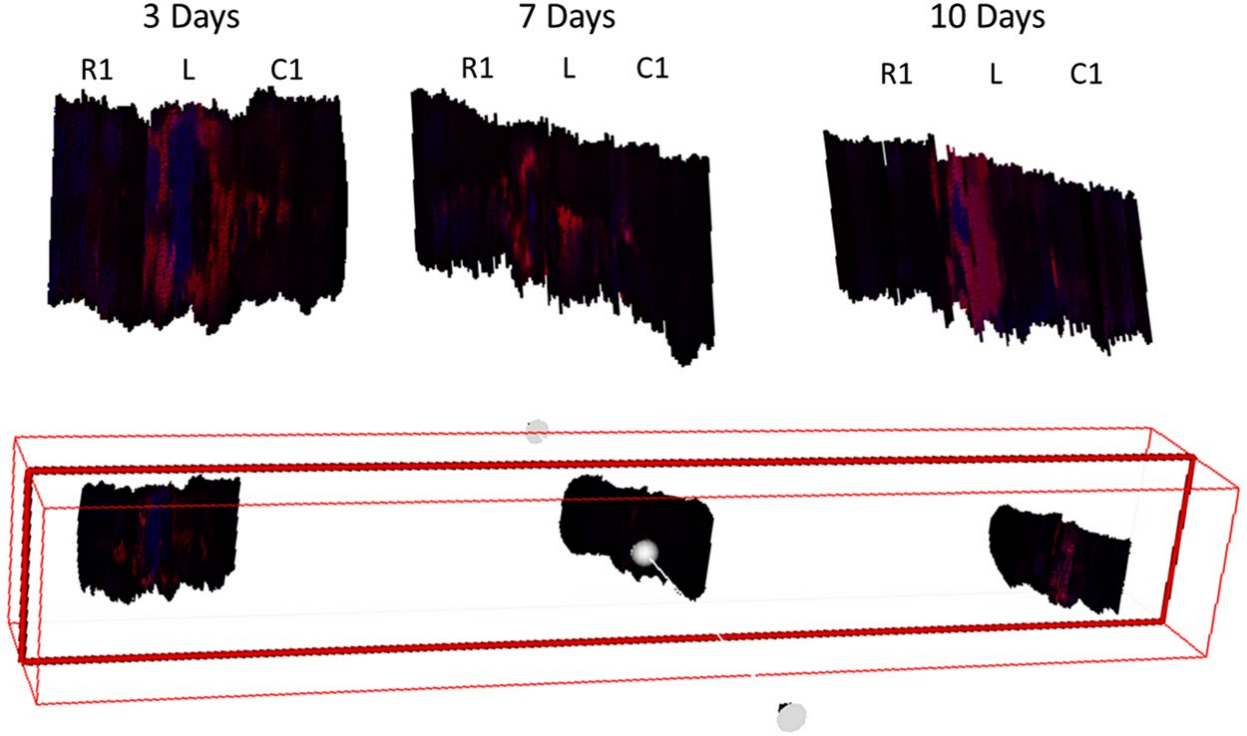
Virtual dissection of the lesion 3, 7 and 10 days after SCI. The plane of dissection (bottom illustration) is parallel to the longitudinal axis of the composite 3D plot of AC(16:0) (m/z 400.3, red) and lysoPC(16:0) (m/z 518.3, blue) for SCI lesion after 3, 7 and 10 days.

In SCI lesion at 3 days, the distribution of AC(16:0) covers the entire length of the lesion extending further into R1 and C1. On the other hand, at 7 (Supplementary Fig. S3) and 10 days (Supplementary Fig. S4) the lesion-specific signals are primarily restricted to the lesion segment except at 10 days for a weak signal in C1. It can also be noted that in sections along the caudal part of L, the distribution of AC(16:0) is interrupted in sections where the outline of the gray matter is discernible. In the 7-day SCI sample, the lipid disruption distribution is difficult to deduce because of the discontinuity of the signal observed in some sections taken from L, although it can be observed that the distribution of AC(16:0) appears to be interrupted also in regions outlined by the gray matter (Supplementary Fig. S3). Virtual dissection along the rostro-caudal axis of the composite 3D reconstructed volume of AC(16:0) and lysoPC(16:0) summarizes these findings (Fig. 4). AC(16:0) outlines the lesion, revealing its turnip shape. The outline is well defined at the rostral part of the lesion and becomes thicker along the caudal part after surpassing the lesion center, which approximately covers the entire girth of the spinal cord. Inside this hollow structure can be found lysoPC(16:0). At 3 days after SCI, the heme-containing fragment of the hemoglobin subunit m/z 616.2 can also be observed within the lesion region. This signal can also be observed in SCI at 7 days but in very weak intensity and in fewer sections only, while in SCI at 10 days it is barely detectable (Supplementary Figs S3, S4).

**Figure 5:**
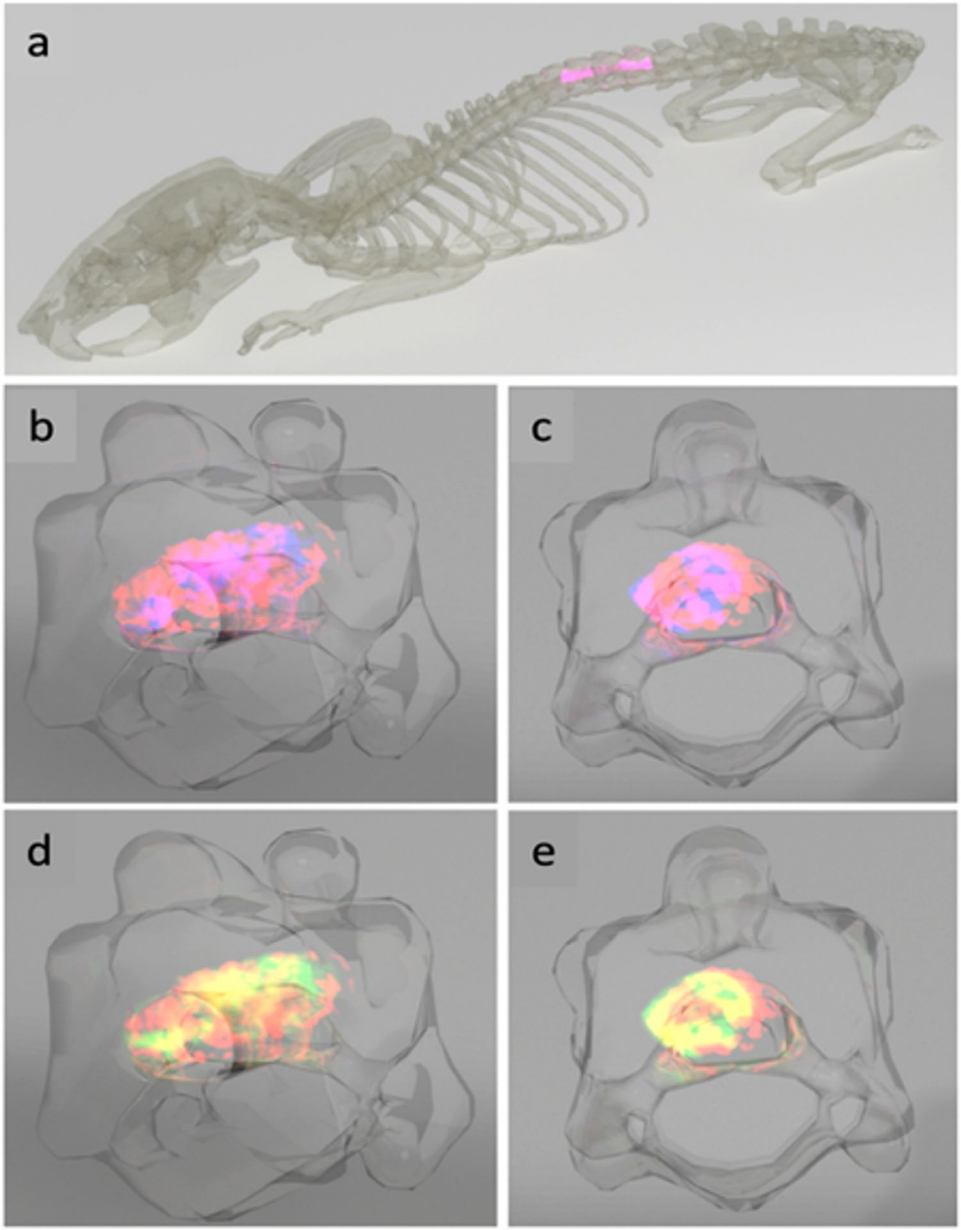
Anatomical rendering of SCI. The spinal cord segments C1, L and R1 were transferred into a rat backbone (**a**) to visualize their localization in the model (pink). The lesion-specific AC(16:0) (m/z 400.3, red) and lysoPC(16:0) (m/z 518.3, blue, (**b**,**c**) as well as AC(16:0) (red) and lysoPC(18:1) (m/z 544.3, green, (**d**,**e**) are shown in a composite 3D plot for the time point 3 days after injury in a side and front view, respectively. Regions where m/z 400.3 and m/z 518.3 and m/z 400.3 and m/z 544.3 overlap appear in pink and yellow, respectively.

The 3D images were then projected in the T7-T9 of the spine of the rat to visualize the lesion and adjacent segments of the spinal cord in the context of their anatomical location (Fig. 5a). Side views of the composite 3D plots of AC(16:0) and lysoPC(16:0) and AC(16:0) and lysoPC(18:1) in the lesion segment show how the turnip-shaped volume outlined by AC(16:0) (red) appears along T8 and encasing both lysoPC(16:0) (blue) and lysoPC(18:1) (green), as revealed by the overlap of m/z 400.3 with the two masses (pink in Fig. 5b,c and yellow in Fig. 5d,e). Front views of the same projections are shown in Fig. 5c and e, where it can be observed that at the lesion center, the extent of overlap almost covers the entire girth of the spinal cord. A video summarizing the main outcomes of this 3D temporal MS imaging study is shown in Supplementary File 1, using the ion distribution of m/z 844.5 to represent the gray matter and that of m/z 822.6 for the white matter.

### Assignment of lesion-specific lipids using High-Resolution (HR) MS and MS^n^

MS^n^ assignment using an HR MS instrument enables the identification of the lipids involved in SCI. Ions showing specific distribution along the lesion margins (m/z 398.3, m/z 400.3 and m/z 426.4) were identified to belong to the LC AC class of lipids (Table 1 and Supplementary Fig. S5). Other MS_n_ as well as MS/MS assignments using MALDI-TOF/TOF and Q-TOF obtained in this work can be found in Supplementary File 2.

**Table 1:** Medium- and long-chain acylcarnitine assignments specific to the lesion site 3, 7 and 10 days after SCI.

R	Common Name	m/z	
14:1	Tetradecanoylcarnitine	372.3	
16:0	Palmitoylcarnitine	400.3	
16:1	Palmitoleoylcarnitine	398.3	
18:1	Elaidic carnitine	426.4	

### Long-chain acylcarnitines co-localize with resident microglia/macrophages found at the lesion margin

**Figure 6:**
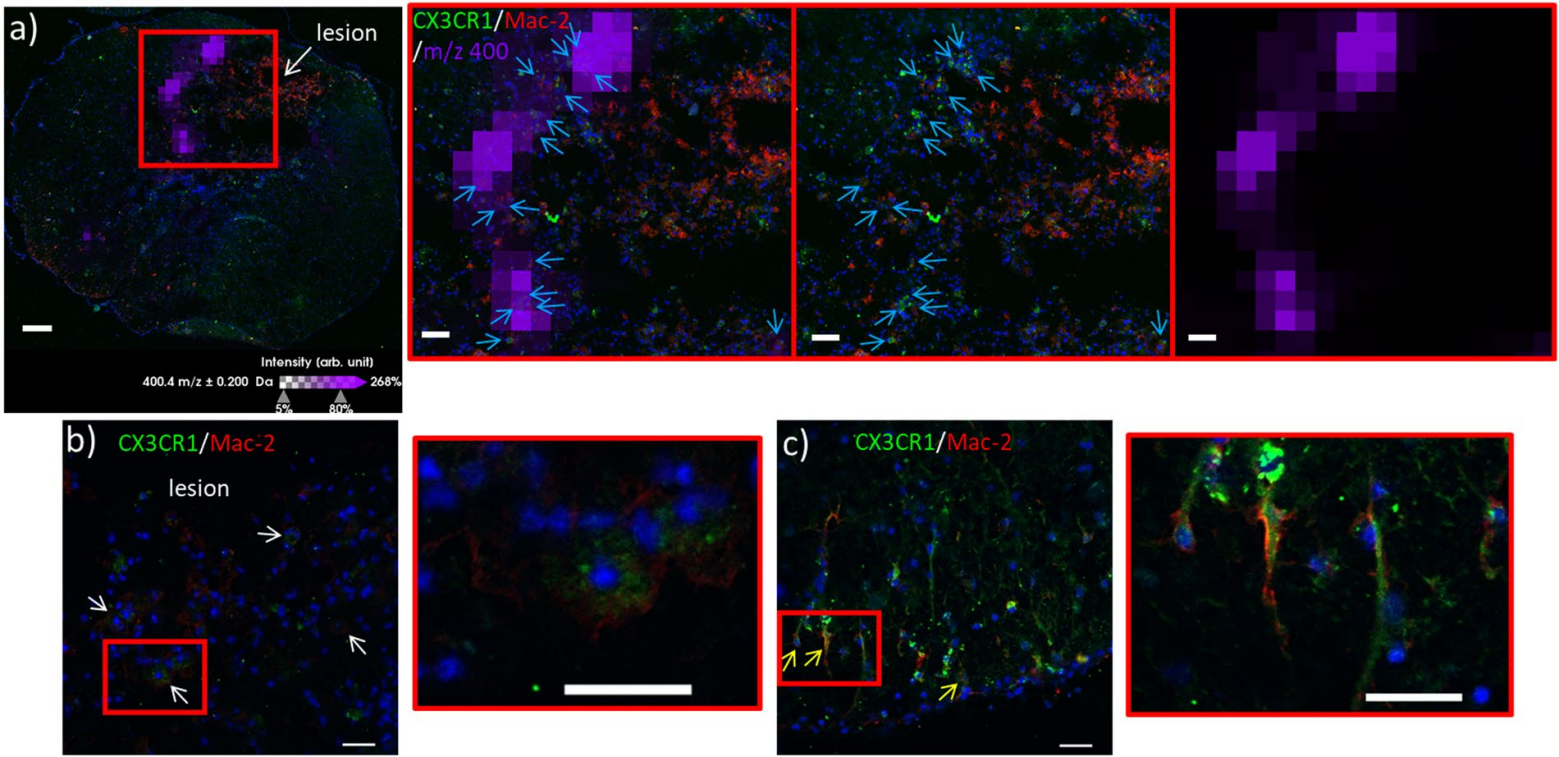
(**a**) Co-registration of the ion image of palmitoylcarnitine AC(16:0) (m/z 400.3, violet) with the mosaic of bright field images of the spinal cord section taken 10 days after SCI and immunostained against CX3CR1 (green) and Mac-2 (red). CX3CR1^+^/Mac2^++^ bone marrow-derived macrophages (BMDMs) coming from blood infiltration are readily observed in the lesion site because of their intense staining against Mac-2. Along the lesion border, resident microglia/macrophages are observable due to their CX3CR1^+++^/Mac-2^+/−^ stain (inset, middle, indicated by light blue arrows). These microglia co-localize with the m/z 400.3 signal (inset, left). In this tissue section, the signal of m/z 400.3 is distinct at the lesion border and absent within the lesion (inset, right). Red box indicates zoomed region. (**b**) Examples of CX3CR1^+++^/Mac-2^+/−^ resident microglia/macrophages observed along the lesion margin (white arrows) at higher magnification, exhibiting hypertrophy with retracted processes (inset), distinct from those observed in regions of the tissue section outside of the lesion site (d, yellow arrows) that show extended processes (inset). Scale bars: a = 100 μm, inset = 70 μm, b and c and insets = 50 μm.

The presence of LC ACs along the lesion margins only could indicate attempts to sequester the injury. Hines *et al*. have previously demonstrated that resident microglia are able to perform this sequestration in laser-induced lesions by extending filopodia and covering the lesion entirely^12^. Ren and co-workers have recently demonstrated that these are macrophages derived from resident microglia, and distinguished them from bone marrow-derived ones (which penetrate the lesion interior instead) by their distinct responses to CX3C chemokine receptor 1 (CX3CR1) and beta-galactoside-binding S-type lectin galectin-3 (Mac-2) immunostaining^13,14^. We thus performed immunostaining of CX3CR1 and Mac-2 and co-registered the images with those from MSI. In order to reconstruct the entire image of the spinal cord section, a mosaic of bright field images was acquired using a confocal microscope, and the entire mosaic was reconstructed and co-registered with the ion image of m/z 400.3. Results revealed that CX3CR1^+++^/Mac-2^+/−^ type staining corresponding to resident microglia is present in the border of the lesion (Fig. 6a and insets). CX3CR1^+/−^/Mac-2^++^ type staining corresponding to BMDMs is detected in the core of the lesion. Along the lesion margin, cells exhibiting CX3CR1^+++^/Mac-2^+/−^ staining co-localize with m/z 400.3 ion distribution (Fig. 6a insets). Reactive microglia exhibit distinct morphology and are hypertrophic with retracted processes along the lesion margins (Fig. 6b and inset). In contrast, microglia detected in regions of the tissue unaffected by the lesion exhibit a ramified morphology with extended processes, indicating that they are in a resting state (Fig. 6c and inset).

## Discussion

In this work we describe using 3D MS imaging techniques the distinct localization to the lesion site of lipids whose abundances are enhanced 3, 7 and 10 days after SCI. Among these are the LC ACs which are intermediates in the acylcarnitine/carnitine shuttle of free fatty acids to the inner mitochondrial membrane during β-oxidation. Accumulation of LC ACs can be a result of mitochondrial damage leading to decreased viability. The LC ACs themselves are pro-inflammatory, and together with medium chain ACs has been demonstrated in insulin resistance and type 2 diabetes mellitus to induce cytokine production and activate cyclooxygenase-2 (COX-2), as well as induce phosphorylation of JNK and ERK via the downstream effector MyD88, and even contribute to ROS production via undeduced mechanisms in RAW 264.7 cells^15^. In addition, the LC ACs have also been shown to exhibit neurotoxicity, and have been implicated in myelinated axon degradation when released by mitochondria-aberrant Schwann cells in the peripheral nervous system (PNS)^16^. Recently, it has also been shown in the context of obesity that LC ACs are capable of activating a reporter gene critical to NF-κB signaling, and indicating that LC ACs could probably perform other functions in relation to chronic inflammation^17,18^. This is further supported by the fact that LC ACs have restricted cellular localization than short chain ACs such as acetyl carnitine, which diffuse more freely in the cell membrane and surprisingly exhibits anti-inflammatory, neurite outgrowth-promoting and neuroprotective effects^19^.

The specific distribution of LC ACs in different areas of the lesion could hint at a well-partitioned immune response. Like LC ACs, lysoPCs are also pro-inflammatory. However, lysoPCs are derived directly from PCs via phospholipase A2 (PLA2)-catalyzed hydrolysis; the latter can be induced during SCI^20^. Mitochondrial dysfunction can also be induced during SCI; however, the distinct distribution of accumulated LC ACs only along the lesion border suggests that only a specific population of cells, albeit localized in this region, is affected. Further experiments are needed to confirm whether the LC ACs are produced by the co-localized resident microglia/macrophages themselves in response to the injury after their recruitment^12^. Nonetheless, the presence of pro-inflammatory LC ACs in this localized microenvironment is one barrier that can possibly lead to aberrant polarization of resident microglia from the M2 to the M1 phenotype^13^, and consequent abrogation of their anti-inflammatory function.

The turnip-shaped distribution of LC ACs along the lesion periphery confirms that the immune response along the rostro-caudal axis is heterogeneous^6^. Rostral to the lesion site where the LC AC and lysoPC distribution is distinct, the microenvironment is neurotrophic and the spinal cord continues to receive brain-derived factors promoting recruitment of T regulator lymphocytes to promote neurite outgrowth and axonal repair^6^. Caudal to the lesion site, where the distinct LC AC and lysoPC distribution is disrupted, the microenvironment is predominantly inflammatory and marked by the presence of neutrophils and absence of neurotrophic signals. Nevertheless, it also has to be considered that the rostral and caudal segments farthest from the lesion site present the same molecular pattern, and with the ability to promote neurogenesis^5^. Inflammation originates from the lesion and spreads to the two adjacent segments (R1 and C1) with a clear distinct pattern^6^. Within a 12-day SCI time course, this pattern of regionalization can be observed as early as 24 h^3^, and is in line with microglia, macrophage and neutrophil recruitment followed by T regulator cells. In fact, as we recently demonstrated, transcription factors from the Smad family involved in neurite outgrowth promotion only arrive after 24h in DRG cells^3^. Inflammation started to propagate from the lesion site to the caudal segment 1–3 days after SCI, which is in line with ROS and β-oxidation activation.

Others have previously reported on the use of MSI to examine SCI as well as traumatic brain injuries (TBI), using MALDI, DESI and other ion sources. Setou and co-workers have reported the rapid decrease and eventual disappearance of docosahexanoic acid (DHA)-containing PCs, PC(16:0/22:6) (m/z 844), PC(18:0/22:6) (m/z 872), PC (16:0/20:4) (m/z 820) and PC (18:0/20:4) (m/z 848), and temporary elevation of arachidonic acid-containing PCs, PC (16:0/20:4) (m/z 820) and PC (18:0/20:4) (m/z 848) as well as lysoPC(18:0) by MALDI MSI beginning 1 day post SCI^11,21^. Even though spectral acquisition was performed between m/z 400 and 1,000, analysis focused on the highly abundant signals at m/z 700–900, thus missing the mass range where LC ACs are normally observed^22^. Likewise, Woods and co-workers applied similar MSI analyses on TBI, revealing brain-specific cerebrosides enhanced by injury, but likewise missing the LC AC mass range^23^. On the other hand, Cooks and co-workers applied DESI imaging on contusion SCI models and succeeded in detecting and mapping deprotonated of forms of important lipid peroxidation products such as AA and DHA in negative mode^24^. However, this is the first report on the detection of LC ACs in response to SCI and its distinct distribution along the rostro-caudal axis.

Although LC ACs have been implicated in various chronic inflammatory diseases and including SCI, interpretation of its accumulation as a result of faulty mitochondrial function remains controversial. Here, we demonstrate that in fact, this accumulation can be considered in terms of its spatial context, highlighting the advantage of the use of MSI for its mapping. Inhibition studies and mapping of LC ACs in other models of CNS injuries are underway, to determine the potential of these lipids as candidate targets for SCI therapy.

## Methods

### Reagents

HPLC grade ACN, absolute ethanol (EtOH), methanol (MeOH), chloroform (CHCl_3_) and water (H_2_O), and analytical reagent (AR) grade trifluoroacetic acid (TFA) were obtained from Biosolve B. V. (Dieuze, France). 2,5-dihydroxybenzoic acid (DHB) and lithium chloride (LiCl) were obtained from Sigma-Aldrich (Saint-Quentin Fallavier, France).

### Animals

The study was performed with approval and in accordance to the guidelines of the Institutional Animal Care and Use Committee of the Slovak Academy of Sciences and with the European Communities Council Directive (2010/63/EU) regarding the use of animals in research, Slovak Law for Animal Protection No. 377/2012 and 436/2012 and protocol approval Ro-4081/17-221.

### Experimental SCI procedure

SCI was induced using the modified balloon compression technique in adult male Wistar rats, described previously^25^. Manual bladder expression was performed 2 times a day for 3–10 days after injury. Experimental SCI rats at 3, 7 and 10 days were sacrificed by isoflurane anesthesia followed by decapitation. Pressure expression of each spinal cord was done by injecting sterile saline (10 ml) throughout the vertebral canal along the caudo-rostral axis. Afterwards, each spinal cord was inspected to verify the centering of the lesion at the Th8-Th9 level on the longitudinal axis. The entire spinal cord was divided into 1-cm segments and those that contained the lesion site (L segment Th7-Th11) and the segments rostral (R1 segment, C1-Th6) and caudal (C1 segment, Th12-L6) to the L segment were subjected to MSI (Fig. S1C). The segments were embedded in optimal cutting temperature (OCT) compound (CML, France) and frozen in isopentane cooled with liquid nitrogen then stored at −80 °C until use.

### Maldi MSI

#### Imaging data acquisition

The entire R1, L and C1 segments were cut into 12-μm sections using a cryostat (Leica Microsystems, Nanterre, France). Sections obtained after every 200 μm (approx.) were subjected to MSI. These were mounted on indium tin oxide (ITO)-coated slides and placed under vacuum in a dessicator for 15 min. DHB was used as matrix, and was prepared at a concentration of 20 mg/mL in 70:30 methanol/0.1% TFA in H_2_O. 12 layers of matrix were deposited using SunCollect plus (SunChrom, Friedrichsdorf, Germany) programmed to spray at gradually increasing flow rates from 10 to 50 µL/min.

Lipid imaging was performed on an AutoFlex Speed instrument (Bruker Daltonics, Bremen, Germany) equipped with a FlashDetector^TM^. The instrument was equipped with a Smartbeam™-II laser capable of operating up to 2 kHz and was controlled using FlexControl 3.3 (Build 108) software (Bruker Daltonics). The datasets were recorded in positive reflector mode and 500 laser shots were accumulated for each raster point. The laser focus was set to medium, and deflection of masses was deactivated. Spectra were acquired at a lateral resolution of 40 μm. External calibration was performed using the PepMix standard (Bruker Daltonics). Spectra were acquired between m/z 300–1100.

#### 2D Image processing and data analysis

The MSI datasets were analyzed with SCiLS Lab 3D, version 2016b (SCiLS, Bremen, Germany)^26^. The baseline was removed by iterative convolution and the data were normalized based on the total ion count (TIC) method^27^. Subsequently, orthogonal matching pursuit (OMP) was used to detect peaks; these peaks were aligned to the mean spectrum by centroid matching and automatic spatial segmentation was performed by using the bisecting k-means algorithm^28^.

A first overview of the spatial regions of white and gray matter was achieved by applying hierarchical clustering using MSI data from all three segments simultaneously. The depth of clustering was selected interactively. Co-localized m/z values with the lesion, white matter and gray matter regions were determined by using Pearson’s correlation analysis^29^. Receiver Operating Characteristic (ROC) analysis was performed to detect m/z values which discriminate between the R1/C1 segments versus the L segment, and L segments at different time points (3 days vs 7 days, 7 days vs 10 days and 3 days vs 10 days)^30^. For the first analysis, a random subset of spectra taken from both R1 and C1 was used to allow the comparison of similar groups of spectra.

#### 3D Image construction, volume visualization and rendering on rat anatomy

For 3D image construction, the MS images were acquired from 12-µm sections taken after cutting about 16 sections from the segment. This resulted in about 36 different sections per segment. Thus, the 3D model was generated out of 36 different sections per segment (R1, L and C1) for all three time points resulting in a model with 324 sections altogether. Registration of different tissue sections was done interactively with rigid 2D alignment. The registered m/z images were passed into the 3D software suite blender for volume visualization. The used rendering technique is ray tracing, i.e., the light is simulated as it passes different media, and thereby various interactions are considered. Materials are modeled by volumetric particles and an intermediate surface which is usually derived from a binary segmentation function. The analyses also include an anatomical model of a rat. The skeleton and the single vertebrate are taken from a processed X-ray CT dataset originally used for 3D printing^31^. For the volumetric m/z image a scalar density function D (x, y, z) of spatial m/z intensities was used. High m/z values give high emission and low m/z values give low or no emission.

### MS/MS

MS/MS spectra were acquired using off-tissue and direct approaches. The off-tissue approach involves liquid-liquid extraction using the Folch method. A 12-µm-thick spinal cord section was suspended in 60 µL MeOH and to this 30 µL CHCl_3_ and 30 µL H_2_O were added. The mixture was vortexed for 1 min, then centrifuged at 10,000 g for 10 min, with the centrifuge temperature maintained at 4 °C. CHCl_3_ extracts were collected and 1 µL was spotted on a MALDI target and combined with 1 µL of matrix alone or containing 5 mg/mL LiCl. The matrix used was 20 mg/mL DHB dissolved in 70:30 MeOH/0.1% TFA.

MALDI LTQ orbitrap XL (Thermo Fisher Scientifique, Bremen, Germany) and UltraFlex II MALDI TOF/TOF were used for both full scan and MS/MS spectral acquisition. The MALDI LTQ Orbitrap XL is equipped with a commercial N_2_ laser (LTB Lasertechnik, Berlin, Germany) operating at λ = 337 nm with a maximum repetition rate of 60 Hz. The hybrid configuration replaces the heated capillary of the electrospray source with a q00 that sends packets of ions into a linear trap for collision-induced fragmentation, with the fragment ions then being transferred to the orbitrap for high-resolution mass analysis. The maximum energy per pulse was set to 10 µJ. Full scans were acquired at 60,000 resolution (at m/z 400) in the positive mode at the same mass range used during imaging acquisition (m/z 300–1100), by averaging 10 or 20 scans acquired with 2 microscans per step. Acquired gain control (AGC) was turned on to determine the optimal number of laser shots per step. Precursor ion isolation was performed using an isolation window between ±1 and ±4 Da and the fragments scanned with a maximum accumulation time between 120 and 200 ms. External calibration was performed using the ProteoMass MALDI Calibration Kit (Sigma-Aldrich, St. Quentin-Fallavier, France).

On the other hand, the MALDI TOF/TOF instrument is equipped with a smartbeam laser capable of operating with a maximum repetition rate of 200 Hz. Full scans were obtained by post source decay, in positive reflector mode. MS/MS spectra were obtained using the LIFT cell with the precursor ions generated with 1000 laser shots and accelerated to 2 kV, and the fragments with 4000 shots and accelerated to 18.80 kV. The reflector potential was set at 29.50 kV. External calibration was performed using the PepMix 6 calibrant (LaserBiolabs, Sophia-Antipolis, France).

The Synapt G2s (Waters, Manchester, UK) coupled to the SPIDERMASS instrument was used for direct analysis of spinal cord tissue sections^32^. 1 μL 5 mg/mL LiCl was spotted on the section and dried prior to analysis. SPIDERMASS uses a tunable wavelength laser system (Radiant version 1.0.1, OPOTEK Inc., Carlsbad, USA) set at 2940 nm and connected to a 10-ns pulse duration Nd:YAG laser pump (Quantel). The laser system is connected to a biocompatible laser fiber (450 µm inner diameter (ID), length of 1 m, HP Photonics IR Fiber) with a 2-cm focusing lens attached at the end. 5 s irradiation at 2 and 4 kJ/m² induces analyte ablation at the tissue surface. The ablated material is aspirated and transported by a Tygon tubing (2.4 mm ID, 4 mm outer diameter (OD), Akron, USA) directly to the inlet of the mass spectrometer by an atmospheric pressure interface^33^. Spectra were acquired in positive mode with a scan time of 500 ms. MS/MS spectra were acquired by CID, with the precursor ion selection window controlled automatically by the instrument. Each spectrum was acquired by averaging 10 scans with each scan acquired for 1 s.

### Immunofluorescence

Immunohistochemical staining was performed on tissue sections which had served for MSI after removal of the MALDI matrix using EtOH washing. After matrix removal, the sections were immersed in blocking buffer (PBS 1x containing 1% bovine serum albumin, 1% ovalbumin, 2% Triton, 1% NDS, and 0.1 M Glycine) for 1 h. The primary antibodies polyclonal rabbit Anti-CX3CR1 (1:30, Santa Cruz Biotechnology, CliniSciences, Nanterre, France) and mouse Anti Mac-2 (1:30, Euromedex, Souffelweyersheim, France) were diluted with the blocking buffer and applied to the sections except for the negative control where only the blocking buffer was applied. The sections were then incubated overnight at 4 °C. The following day, the sections were washed three times with PBS 1×, and incubated for 1 h at 37 °C with the secondary antibodies Alexa fluor donkey anti rabbit, and Alexa fluor donkey anti mouse (Life Technologies, ThermoFisher Scientific, Courtaboeuf, France) both diluted in blocking buffer without 0.1 M glycine. Afterwards, the sections were further washed with several changes of PBS 1×, stained with Sudan black 0.3% for 10 min in order to decrease the background signal generated by lipids, and were eventually counterstained with Hoechst solution (1:10,000). The slides were then washed with PBS 1×, and Dako fluorescent mounting medium was applied on the sections before putting cover slips. Mosaic widefield images composed of 100–150 image scans of 512 × 512 dpi resolution were obtained using a confocal microscope (Leica Biosystems, Nussloch, Germany). Confocal images were also obtained at a confocal depth of 3 μm.

## Electronic supplementary material

Supplementary Information

3D MSI of injured rat spinal cord

## Data Availability

MS images and the SCiLS file are available upon request from the corresponding author.
