# Supplementary material for: 3D MALDI mass spectrometry imaging reveals specific localization of long-chain acylcarnitines within a 10-day time window of spinal cord injury
Source: Sci Rep. 2018 Oct 31;8:16083. doi: 10.1038/s41598-018-34518-0 (PMC6208337; doi:10.1038/s41598-018-34518-0)

Supporting Information

3D MALDI mass spectrometry imaging reveals specific localization of long-chain acylcarnitines within a 10-day time window of spinal cord injury

Jusal Quanico[a]*, Lena Hauberg-Lotte[b]*, Stephanie Devaux[a], Zahra Laouby[a], Celine Meriaux[a], Antonella Raffo-Romero[a], Melanie Rose[a], Leie Westerheide[b], Jost Vehmeyer[b], Franck Rodet[a], Peter Maass[b], Dasa Cizkova[c,d], Norbert Zilka[d], Veronika Cubinkova[d], Isabelle Fournier[a]**, Michel Salzet[a]**

a INSERM U1192 - Laboratoire Protéomique, Réponse Inflammatoire et Spectrométrie de Masse (PRISM), Université de Lille 1, F-59000 Lille, France

b Center for Industrial Mathematics, University of Bremen, Bibliothekstraße 5, 28359 Bremen, Germany

c Department of Anatomy, Histology and Physiology, University of Veterinary Medicine and Pharmacy in Kosice, Komenskeho 73, 041 81 Kosice, Slovakia

d Institute of Neurobiology, Slovak Academy of Sciences, Dubravska cesta 9, 845 10 Bratislava, Slovakia

Supplementary Tables and Figures

Supplementary Table S1. Co-localized signals obtained by Pearson's correlation analysis.

Lesion Site		Gray Matter		White Matter	
m/z	Corr.	m/z	Corr.	m/z	Corr.
398.3	0.25	307.1	0.39	339.7	0.26
399.2	0.16	592.2	0.37	393.8	0.13
400.3	0.32	610.9	0.32	395.3	0.19
426.4	0.31	683.3	0.36	453.7	0.17
427.4	0.31	709.5	0.35	453.8	0.20
518.3	0.33	756.5	0.41	552.6	0.16
534.3	0.32	757.5	0.38	553.0	0.17
544.3	0.27	772.6	0.44	747.0	0.25
546.3	0.32	773.5	0.39	747.2	0.27
562.3	0.32	829.5	0.21	761.1	0.23
721.6	0.26	830.5	0.39	761.2	0.25
722.5	0.14	844.5	0.49	762.1	0.22
725.5	0.25	845.6	0.45	787.5	0.20
		846.5	0.43	789.1	0.24
		872.5	0.43	789.2	0.26
				813.1	0.09
				813.6	0.20
				814.1	0.16
				815.5	0.20
				816.9	0.14

Supplementary Table S2. Time point-discriminative m/z obtained by ROC analysis of spectra from the L segments. *

Lesion at 3 days	Lesion at 7 days	Lesion at 10 days	
m/z	m/z	m/z	
374.04	1009.455	706.554	779.553
375.073	1025.524	722.508	780.471
375.991		735.478	783.57
389.994		736.511	784.489
396.995		737.544	785.522
412.031		741.447	786.555
413.064		751.547	787.473
427.412		752.465	790.572
443.71		753.498	800.558
551.028		754.531	802.509
566.983		761.533	804.575
589.02		762.566	805.493
604.975		763.484	806.526
620.929		764.517	807.559
		769.567	812.609
		770.486	813.528
		771.519	820.529
		772.552	824.546
		773.47	851.634
		774.503	852.552

* Grouping procedure for detection of time-point discriminative m/z: For SCI lesion at 3 days, ROC analyses using 3 day vs. 7 day and 3 day vs. 10 day comparisons were performed, and m/z common in both analyses with AUCmax > 0.80 (“above”) were assigned as discriminative of the lesion at 3 days. The same approach was used to find discriminative m/z for the 7 day and 10 day time points. For SCI lesion at 7 days, m/z with AUCmax < 0.30 (“below”) from the 3 day vs. 7 day comparison, and those with AUCmax > 0.70 (“above”) from the 7 day vs. 10 day comparison, were used. Whereas, for SCI lesion at 10 days, m/z with AUCmax < 0.30 (“below”) from the 3 day vs. 10 day and 7 day vs. 10 day comparisons were used.

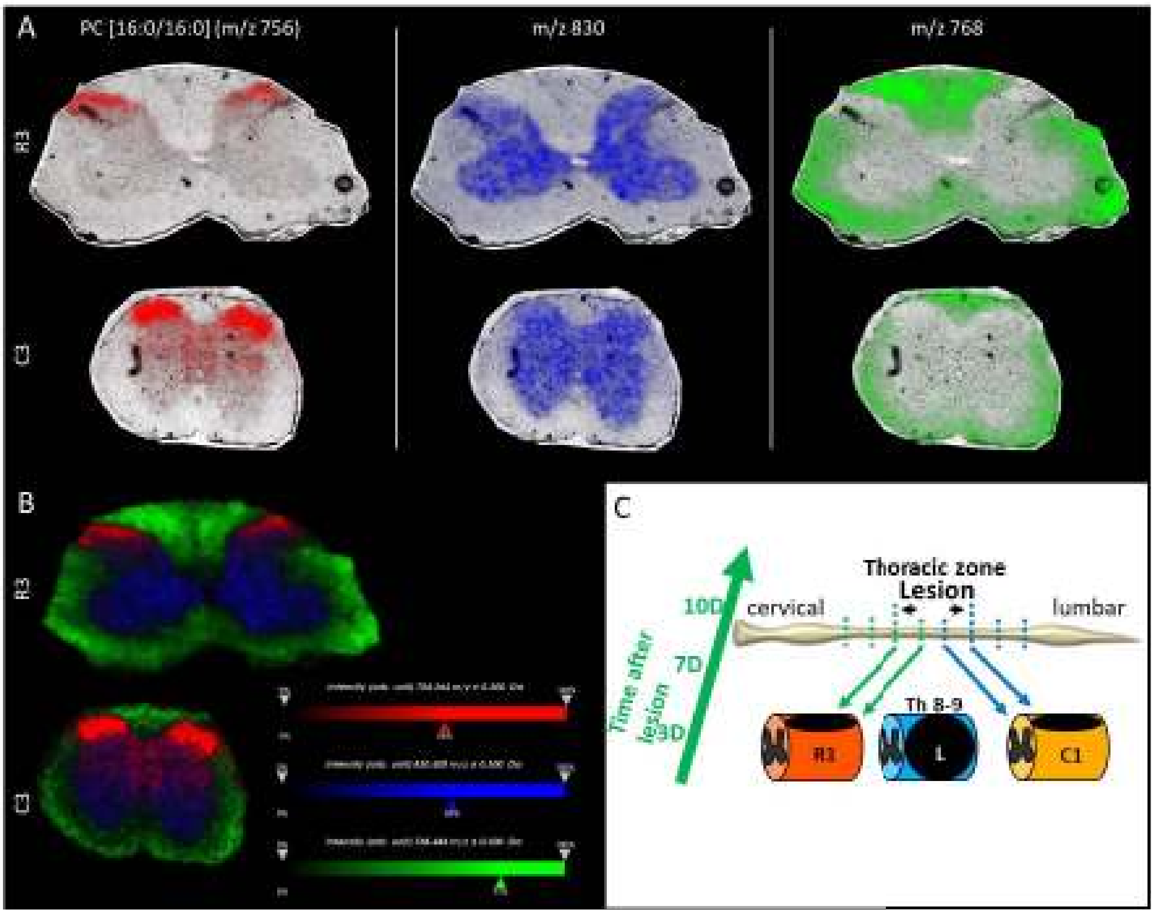

Supplementary Figure S1. MSI of unaffected rat spinal cord. A) Ion images of PC [16:0/16:0], m/z 830.5 and m/z 768.6 in rostral (R3, corresponding to cervical 5-6) and caudal (C3, corresponding to lumbar 6-sacral 1) spinal cord segments taken 3 cm away from the lesion, showing their undisrupted distribution. The images are superposed with optical scans of the imaged sections. B) Composite image of PC [16:0/16:0], m/z 830.5 and m/z 768.6. C) Diagram illustrating the location of spinal cord segments taken for 3D MSI.

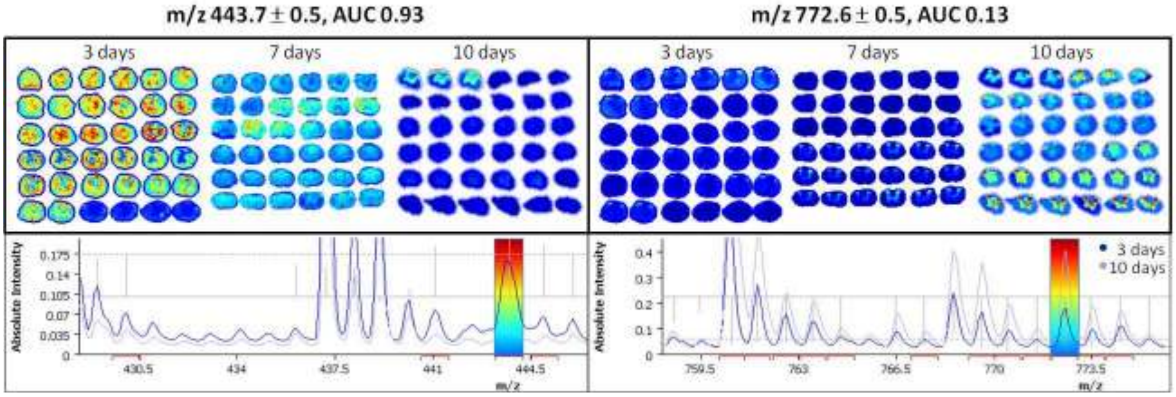

Supplementary Figure S2. Examples of m/z values detected in SCI lesions that are time point-discriminative based on ROC analysis (L at 3 days vs. L at 10 days). Top panels show the ion images of m/z 443.7 (discriminative of L at 3 days, ie., AUC > 0.80) and m/z 772.6 (discriminative of L at 10 days, ie., AUC < 0.30) of all sections from the L segments. Bottom panels show mean absolute intensities of the m/z intervals (highlighted in rainbow) of spectra from all imaged sections in SCI lesions at 3 days (blue) and 10 days (light violet).

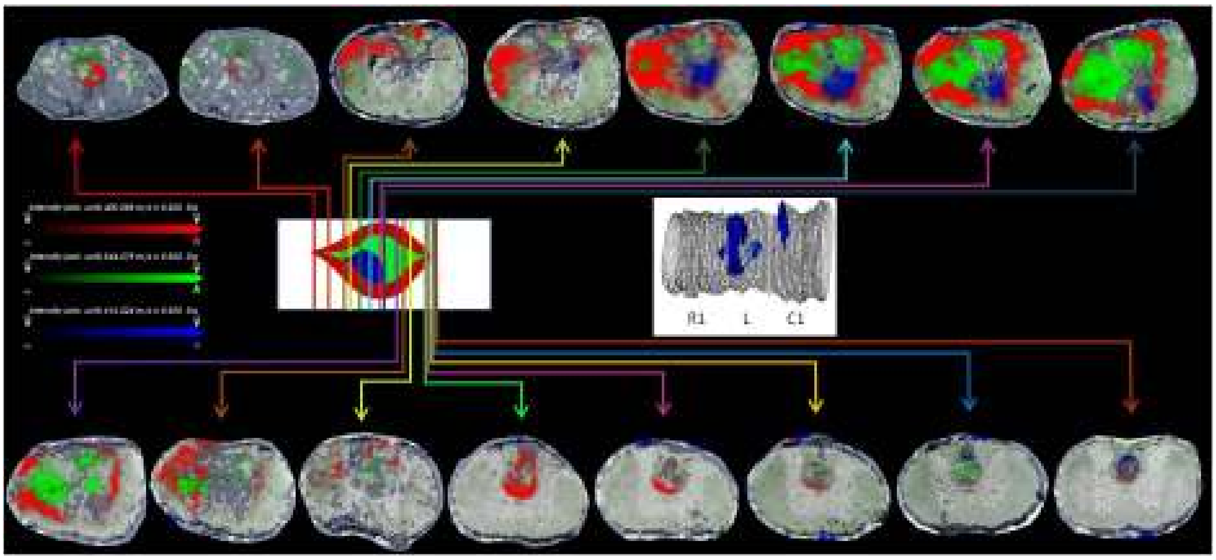

Supplementary Figure S3. Composite images of m/z 400.3 (red), m/z 544.3 (green) and m/z 616.2 (blue) in sections taken at various locations along the rostro-caudal axis of the spinal cord 7 days after injury, as indicated in the idealized cross-sectional diagram of the lesion site. Inset shows the 3D image stack of m/z 400.3 in all sections from segments R1, L and C1 subjected to MSI.

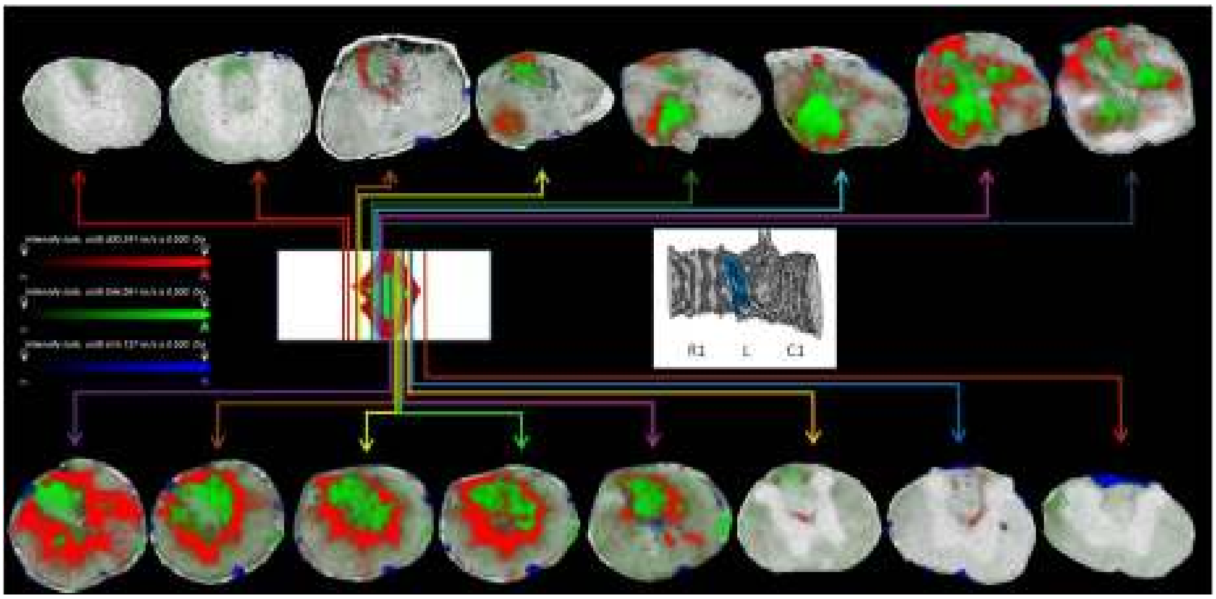

Supplementary Figure S4. Composite images of m/z 400.3 (red), m/z 544.3 (green) and m/z 616.2 (blue) in sections taken at various locations along the rostro-caudal axis of the spinal cord 10 days after injury, as indicated in the idealized cross-sectional diagram of the lesion site. Inset shows the 3D image stack of m/z 400.3 in all sections from segments R1, L and C1 subjected to MSI.

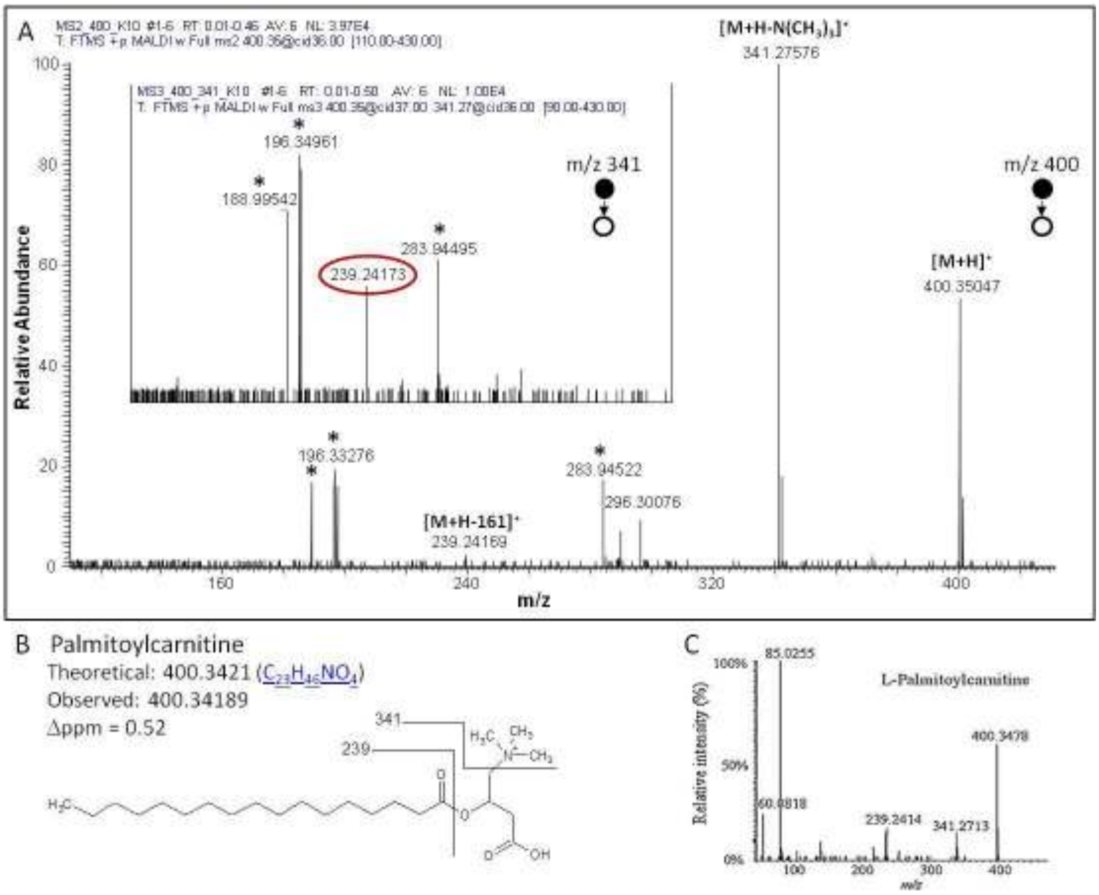

Supplementary Figure S5. Identification of palmitoylcarnitine by MS^n. a) MS2 spectrum of m/z 400.3, showing m/z 341 as the stable fragment ion after loss of trimethylamine. MS3 spectrum of m/z 341 yields m/z 239 (inset). b) Assignment of palmitoylcarnitine and comparison of high accuracy mass measurement. c) MS2 spectrum of L-palmitoylcarnitine obtained using a Quatro QQQ instrument (Lin et al. 2011[10], reproduced with permission from Elsevier). Asterisks (*) denote artifacts due to pump noise, etc., consistently observed in all spectra.

The MS2 spectrum of the lesion-specific peak m/z 400.3 shows the prominent peaks of the precursor ion and the fragment after loss of the N-trimethylamine group (m/z 341, Figure S5a). The peak at m/z 239 corresponding to the loss of the glycerol backbone can also be observed as a weak signal in the MS2 spectrum. MS3 of m/z 341 yields the same fragment, confirming its presence (Figure S5a, inset). Mass matching with the predicted mass of AC(16:0 (m/z 400.3421) further confirms the assignment, with Δppm = 0.52 (Figure S5b). Likewise, Lin et al. obtained the same initial fragments in an MS2 spectrum generated on a Quatro QQQ instrument (Figure S5c)[1]. In the same manner, assignment of AC(18:1) (m/z 426.4) was done, with Δppm = 0.56. For AC(18:1), the fatty acid fragment after loss of glycerol (m/z 265) is already observable in MS2 (Supplementary Data 3). In the case of m/z 398.3, isolation of the precursor ion proved to be difficult. However, LIPIDMAPS database interrogation of the high-accuracy mass measurement (m/z 398.32619) suggests this mass to be AC(16:1) (Δppm = 0.78), the mono-unsaturated form of AC(16:0). m/z 398.3 is also lesion-specific and co-localizes with m/z 400.3. m/z 372.3 was also identified as another acylcarnitine member, AC(14:0) (Δppm = 1.80); in this case only the N-trimethylamine and –COOH losses were observed. m/z 372.3 was identified as lesion-discriminative by ROC analysis. Peaks co-localized in the gray matter and white matter regions were also assigned, where possible; following lithium adduct formation a (Supplementary File 2).

Supplementary File 2. Annotated MS2 and MS3 spectra of lipid species detected in spinal cord after injury.

Asterisks (*) mark background signal observed in all MSn spectra

Elaidic carnitine
Cn-(18:1)

Theoretical Mass: 426.3578 ($C_{25}H_{48}NO_4$)
Measured Mass: 426.35756
Δppm = 0.56

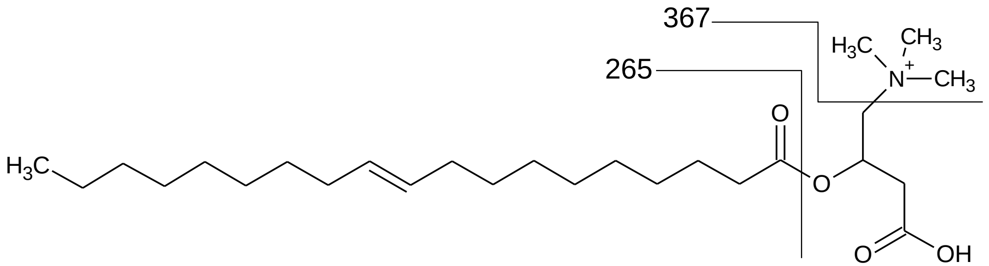

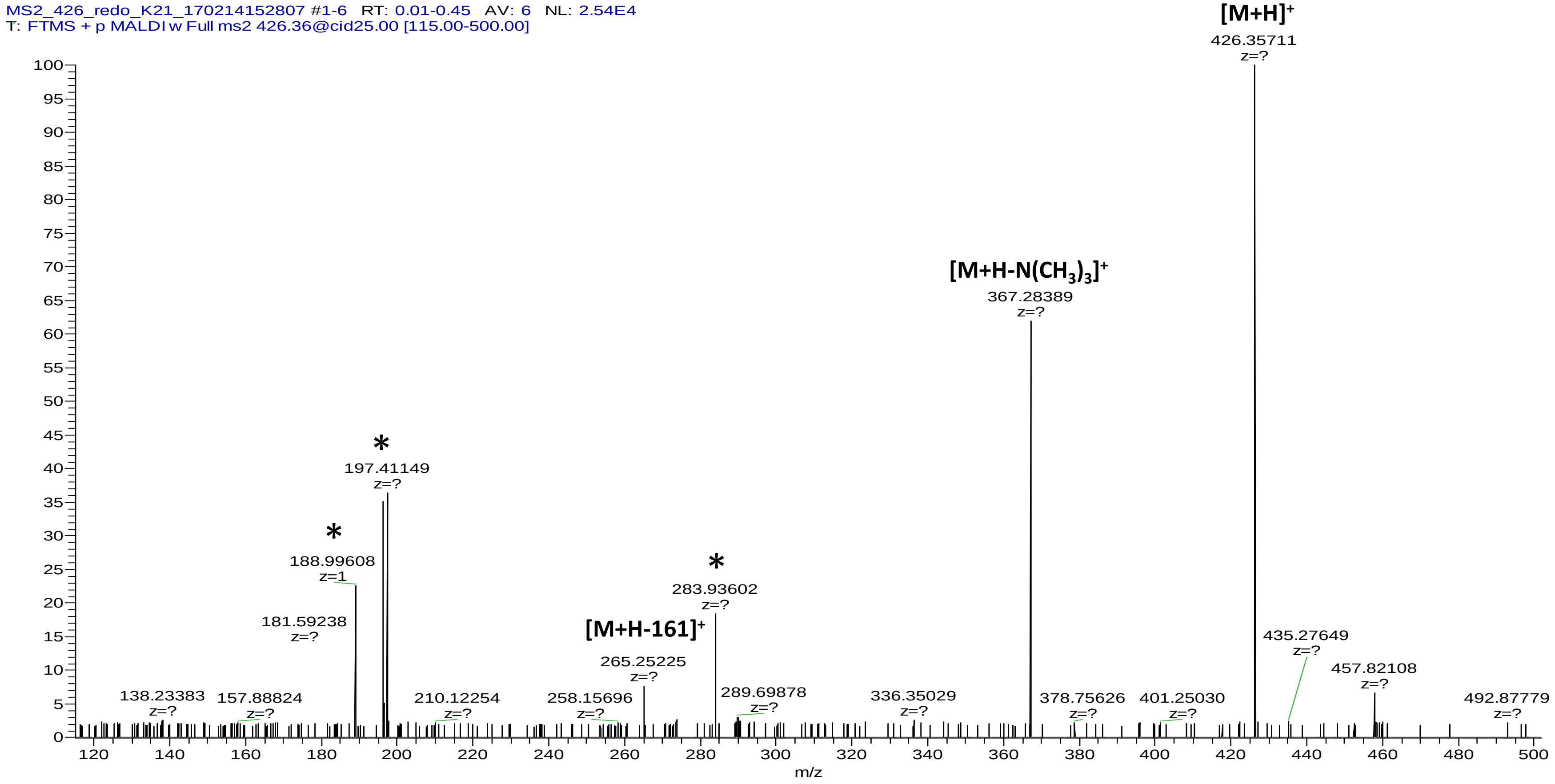

Tetradecanoylcarnitine
Cn-(14:0)

Theoretical Mass: 372.3108 ($C_{21}H_{42}NO_4$)
Observed Mass: 372.31013
Δppm = 1.80 ppm

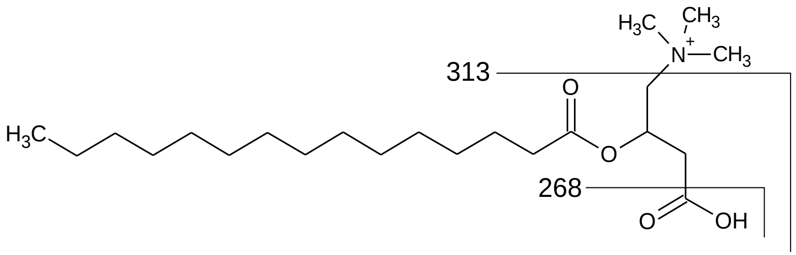

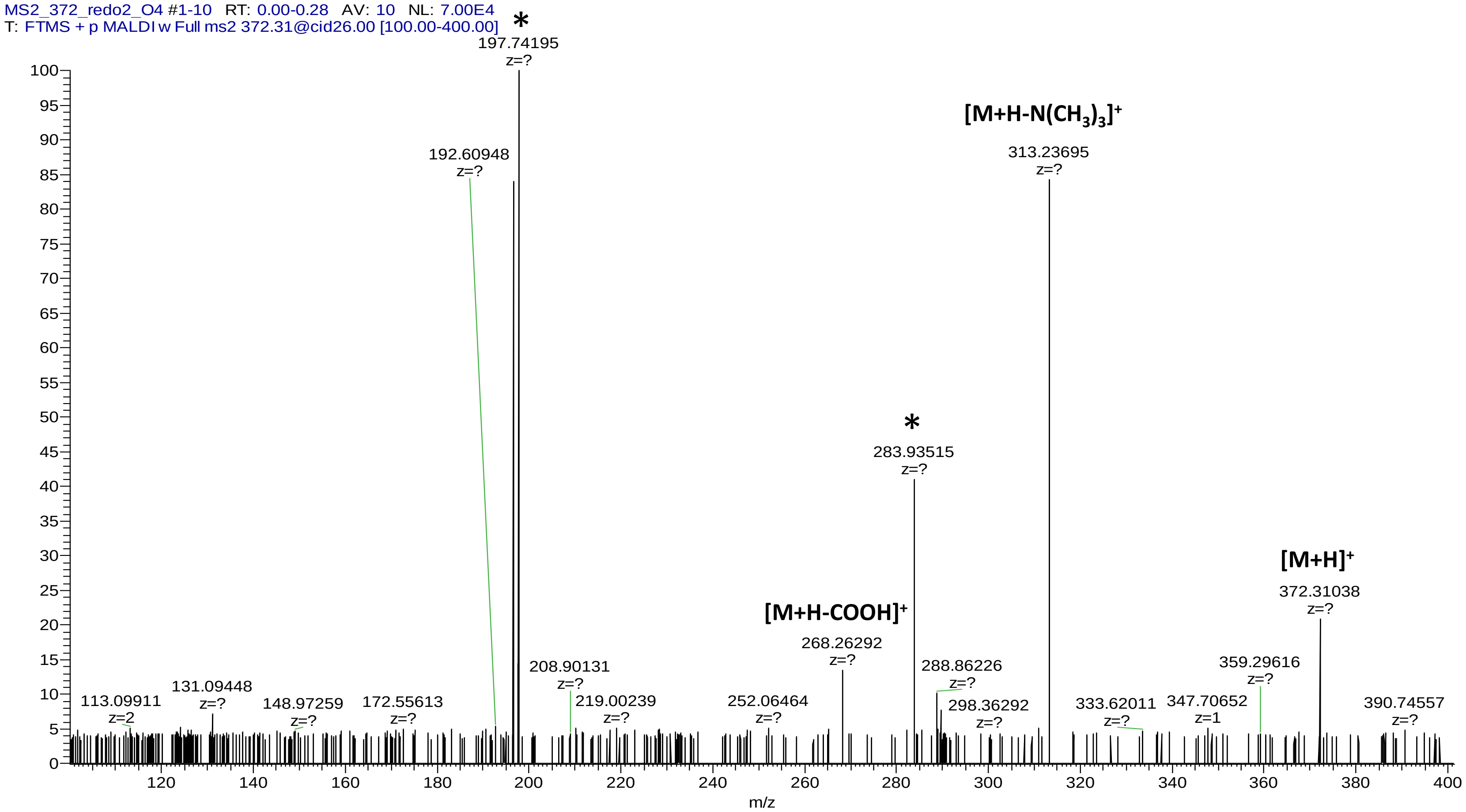

lysoPC(16:0)

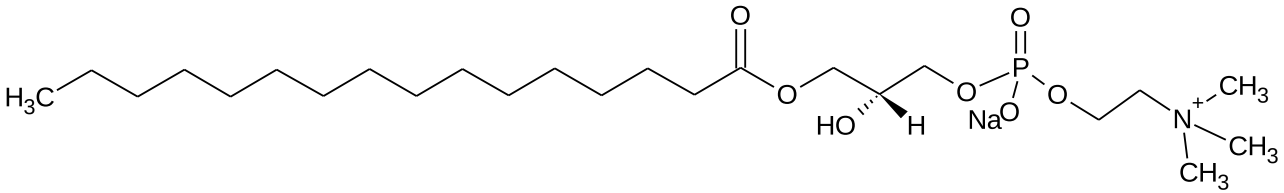

Theoretical Mass: 518.3217 ($C_{24}H_{50}NNaO_7P$)
Measured Mass: 518.32138
Δppm = 0.62

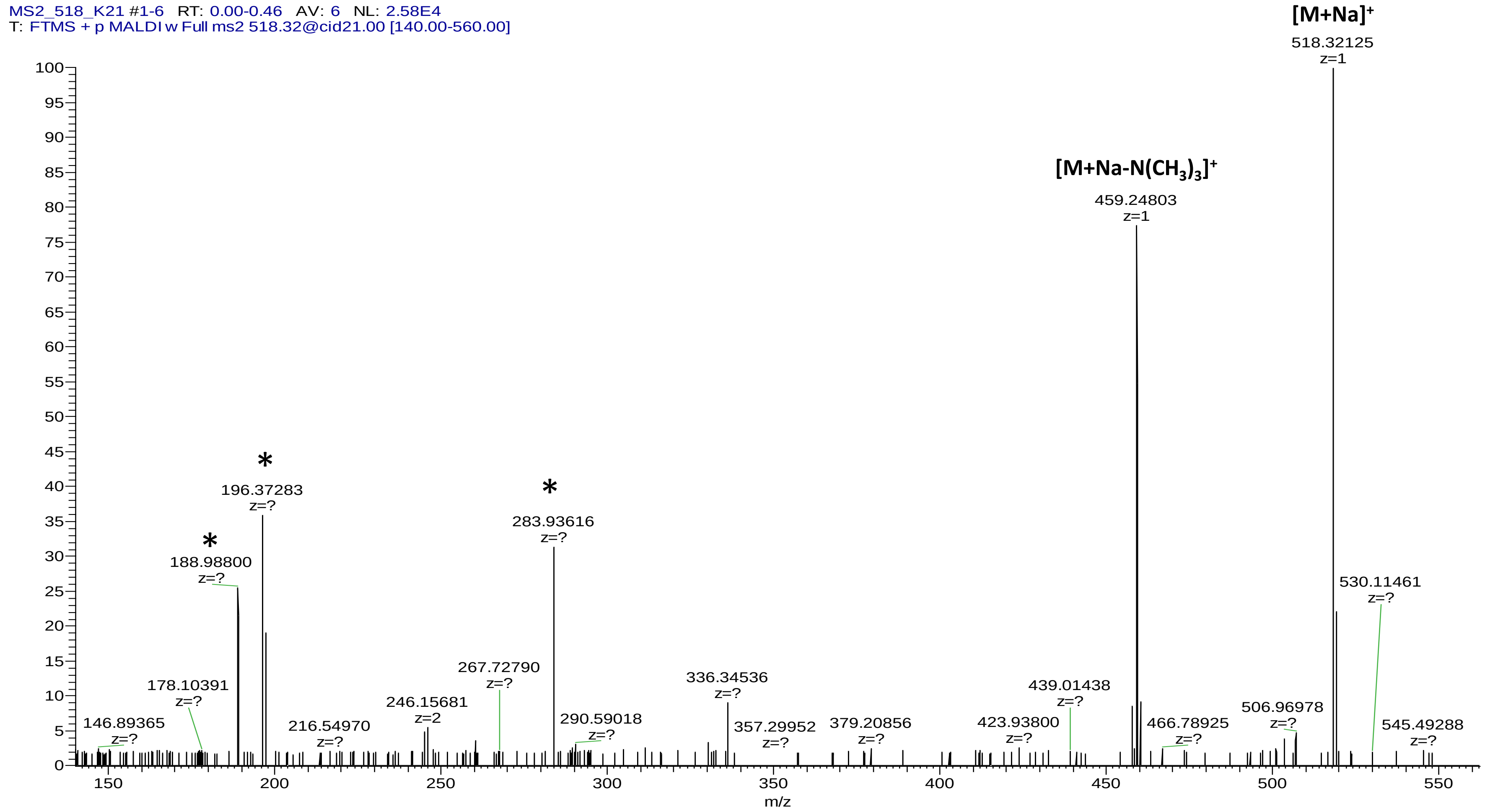

lysoPC(18:0)

Theoretical Mass: 524.3711 ($C_{21}H_{42}NO_4$)
Observed Mass: 524.36999
Δppm = 2.12 ppm

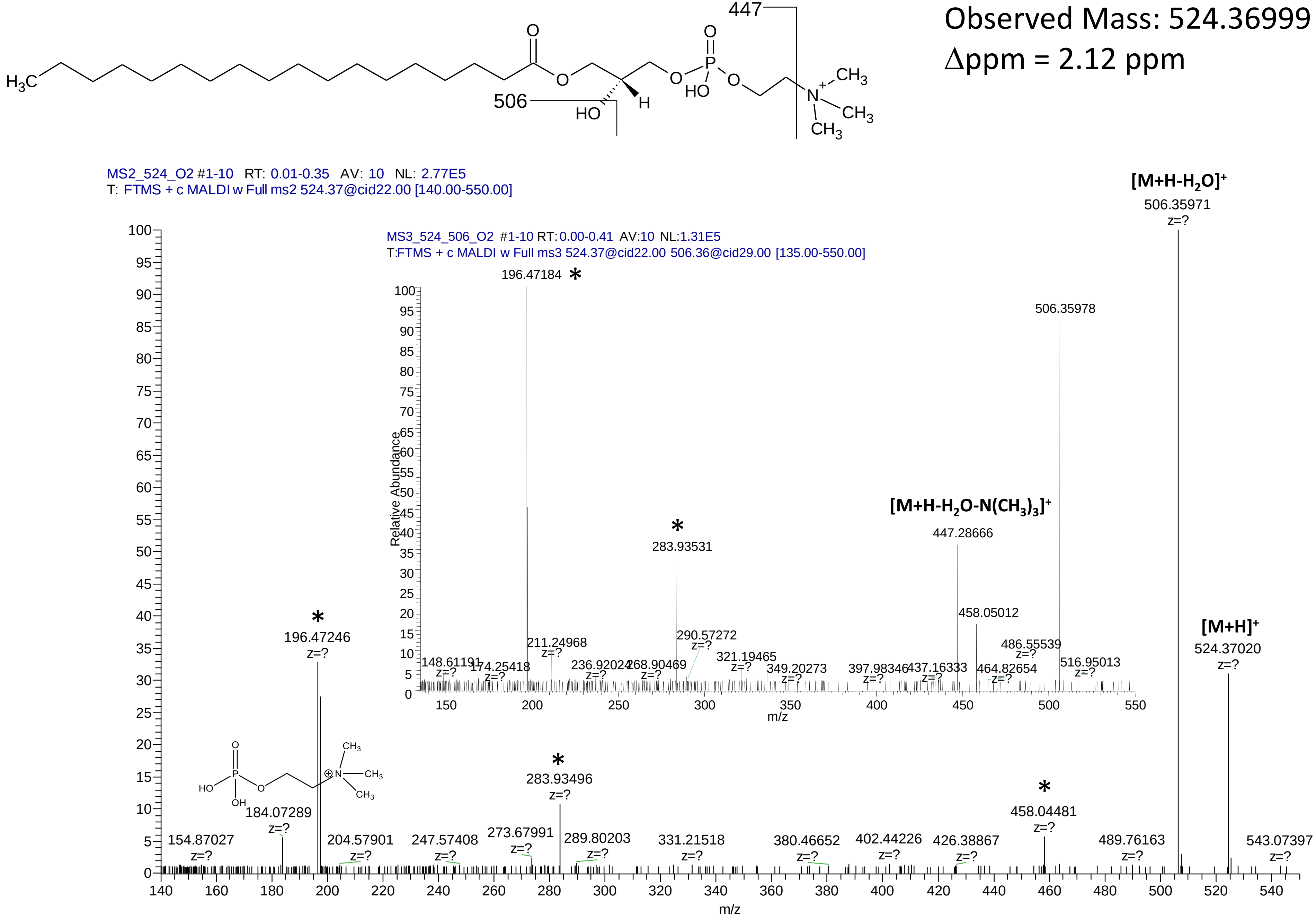

lysoPC(18:0)

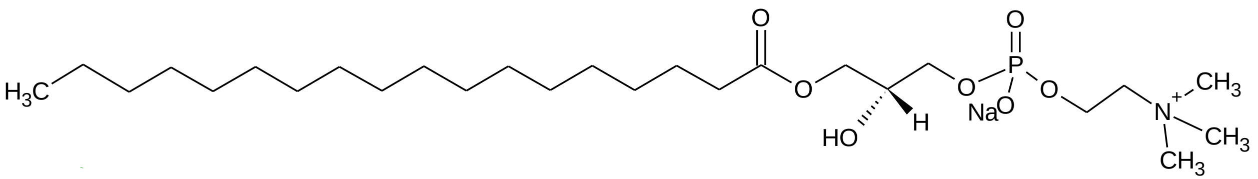

Theoretical Mass: 546.3530 ($C_{26}H_{54}NO_7PNa$)

Observed Mass: 546.35261

Δppm = 0.71

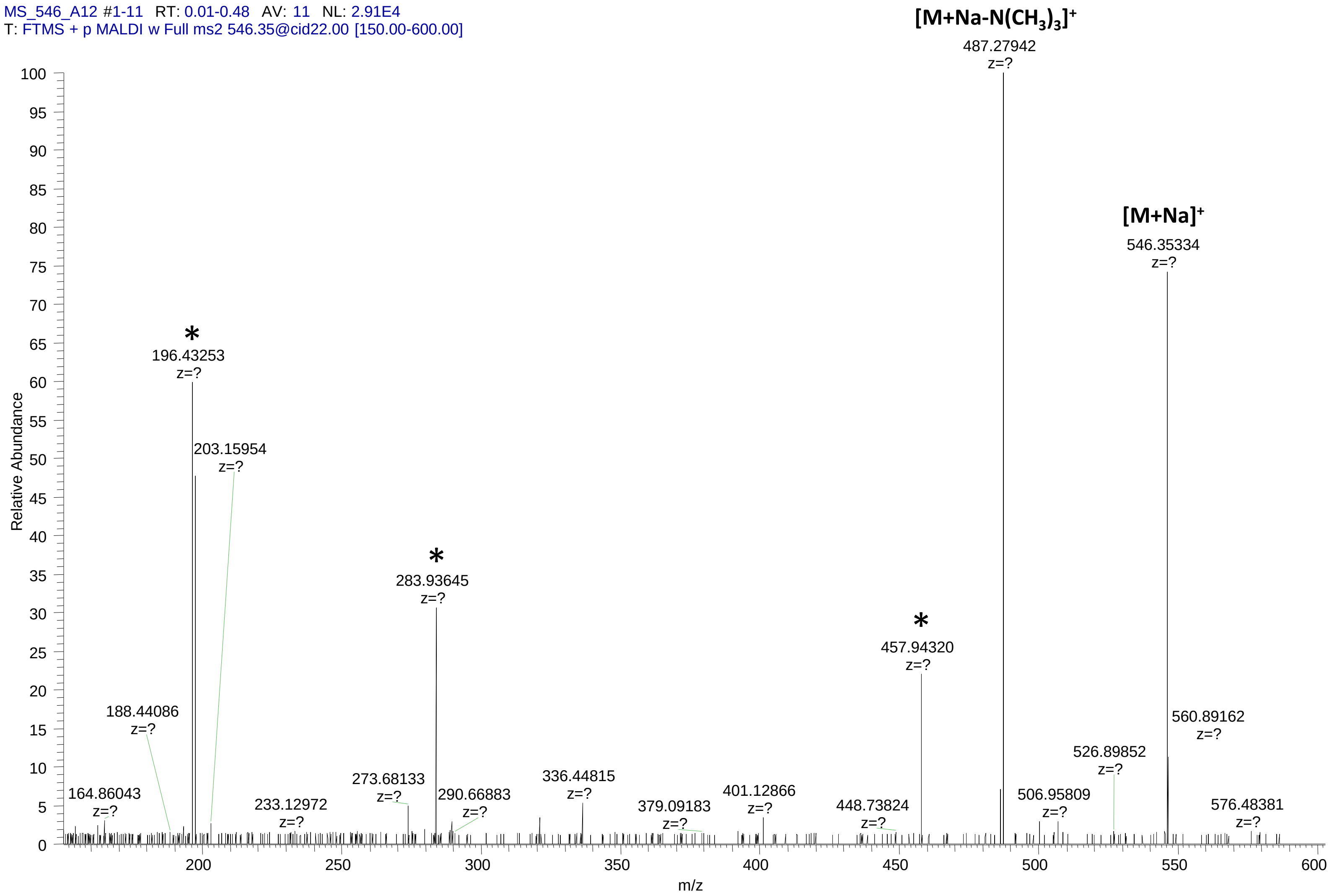

lysoPC(18:1)

Theoretical Mass: 544.3374 ($C_{26}H_{52}NNaO_7P$)

Observed Mass: 544.33851

Δppm = 2.04 ppm

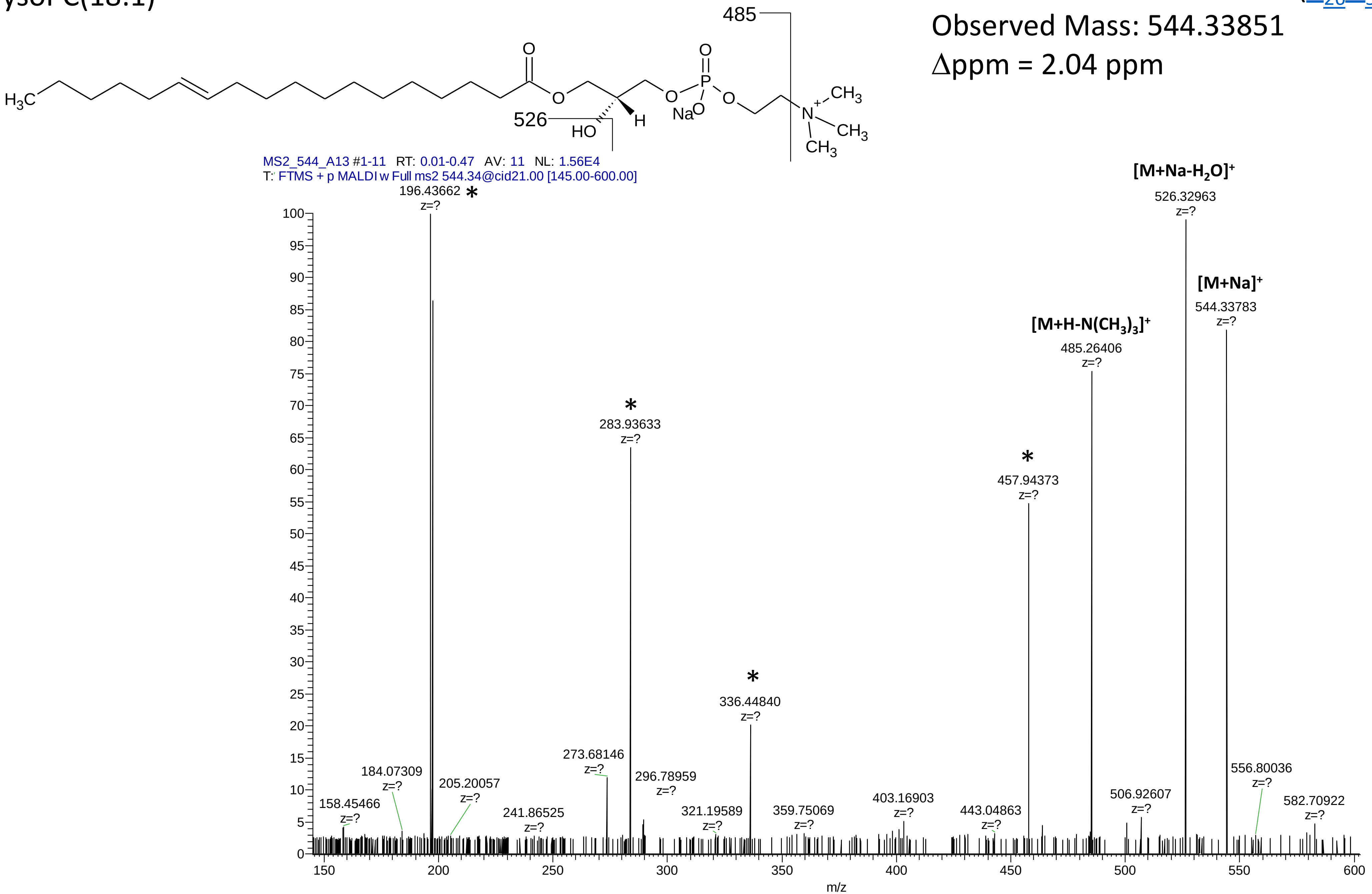

26:1(9OH,10OH)

[M+H]+ 427

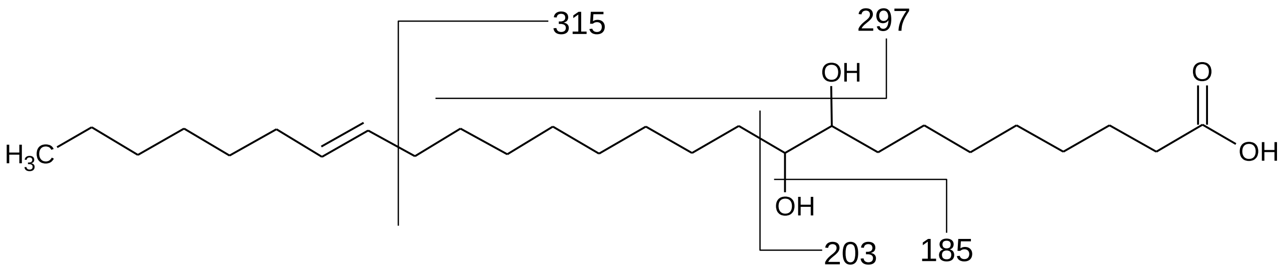

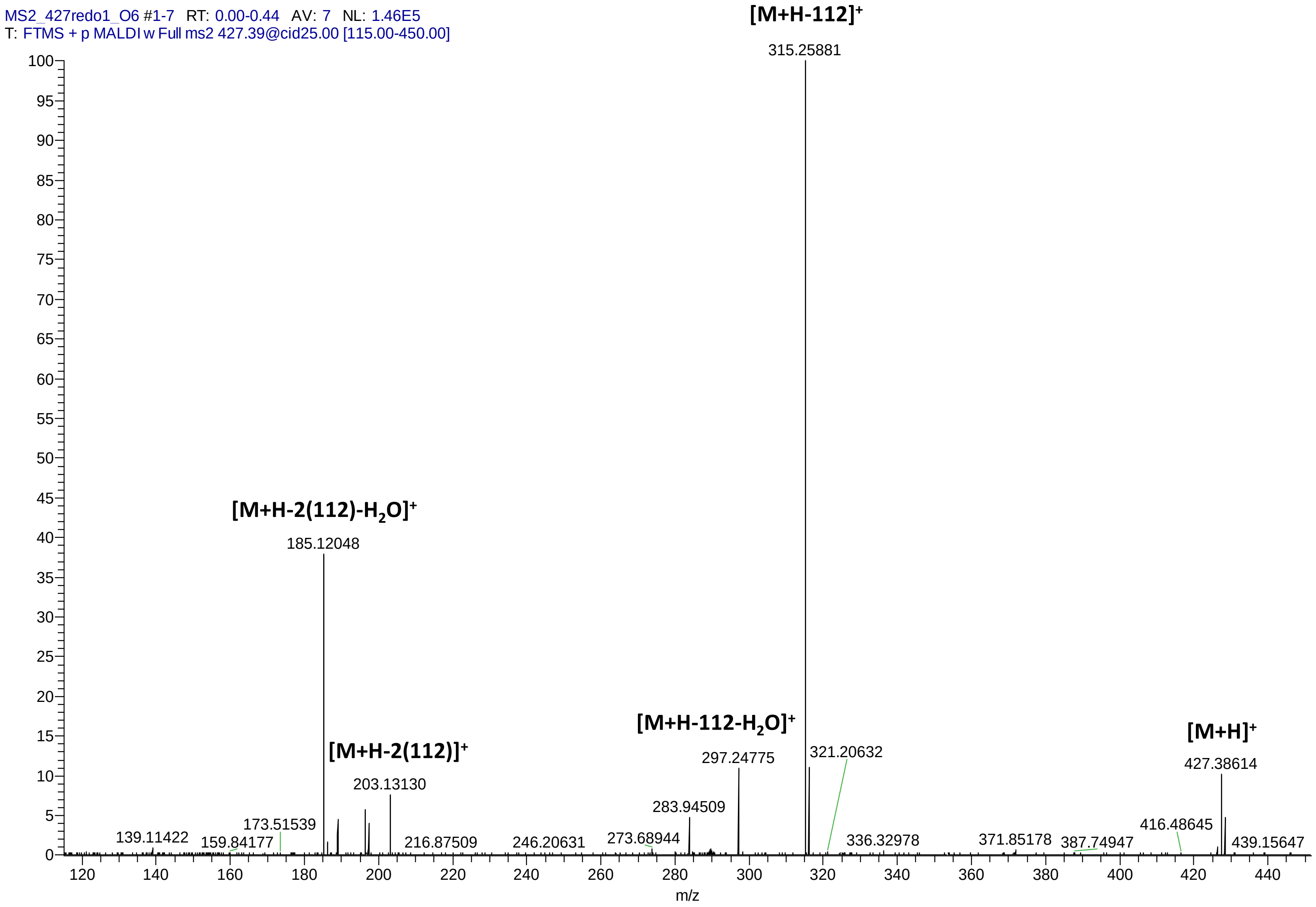

PC (16:0/16:0) Dorsal horn-specific

Theoretical Mass: 756.5514 ($C_{40}H_{80}NO_8PNa$)
Observed Mass: 756.553329
Δppm = 2.31 ppm

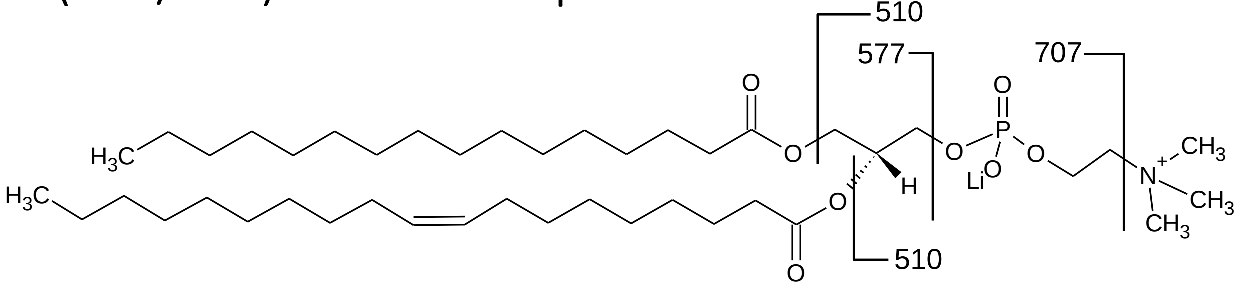

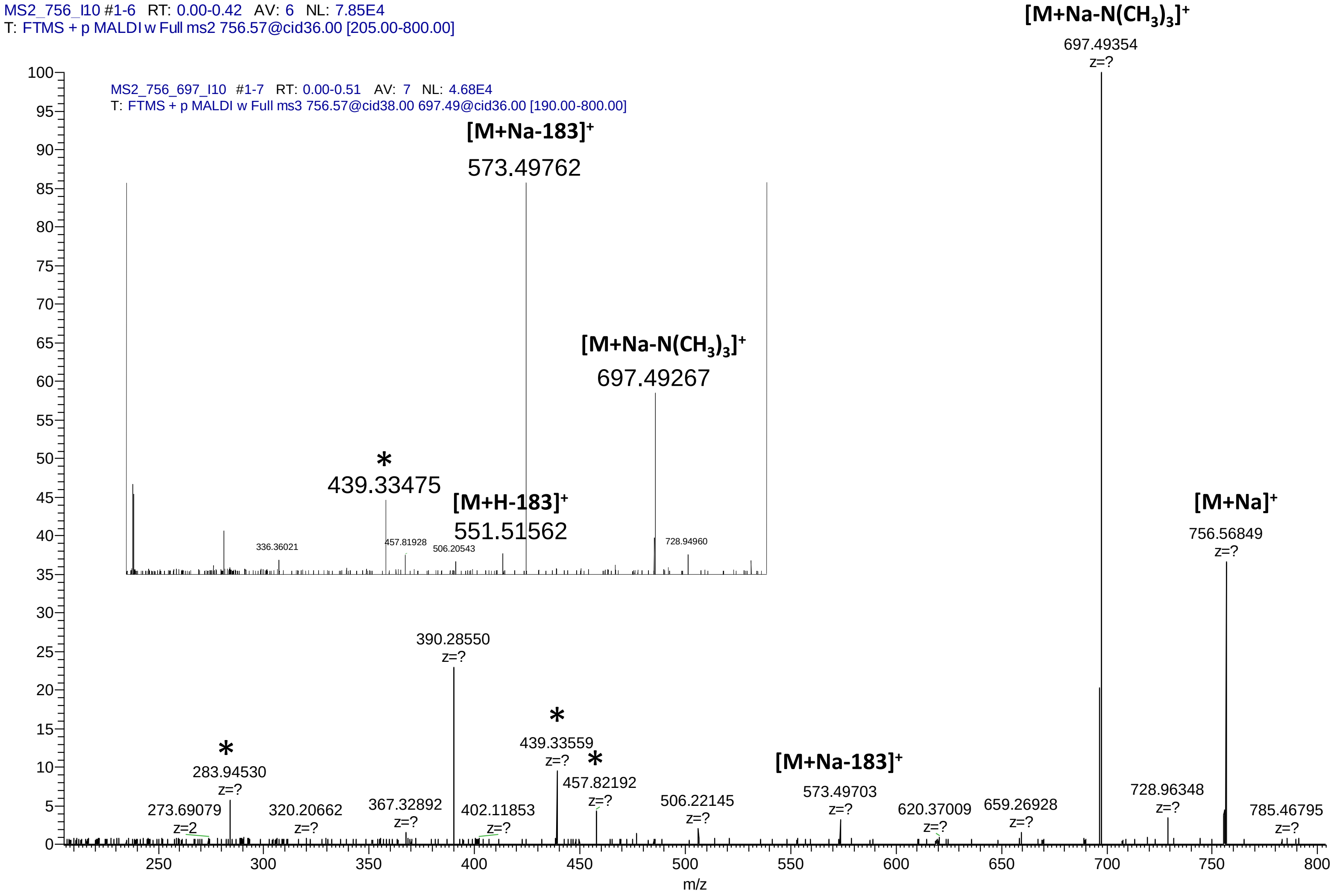

PC (16:0/18:1) White Matter-specific PC

[M+H]+ 760, [M+Li]+ 766

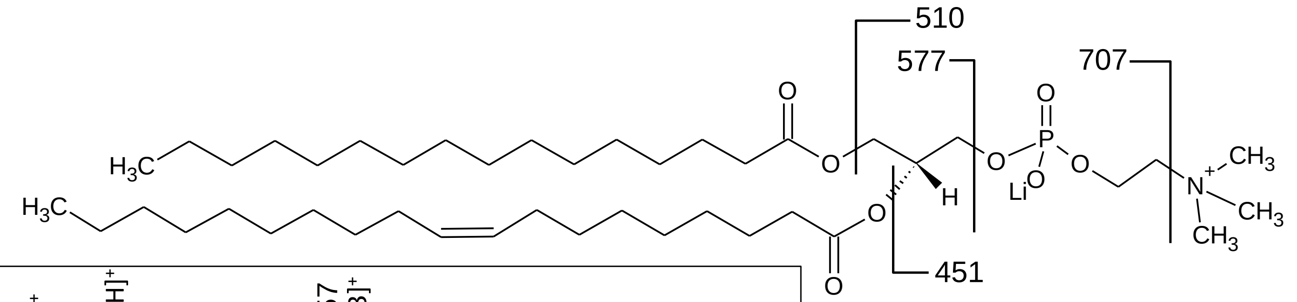

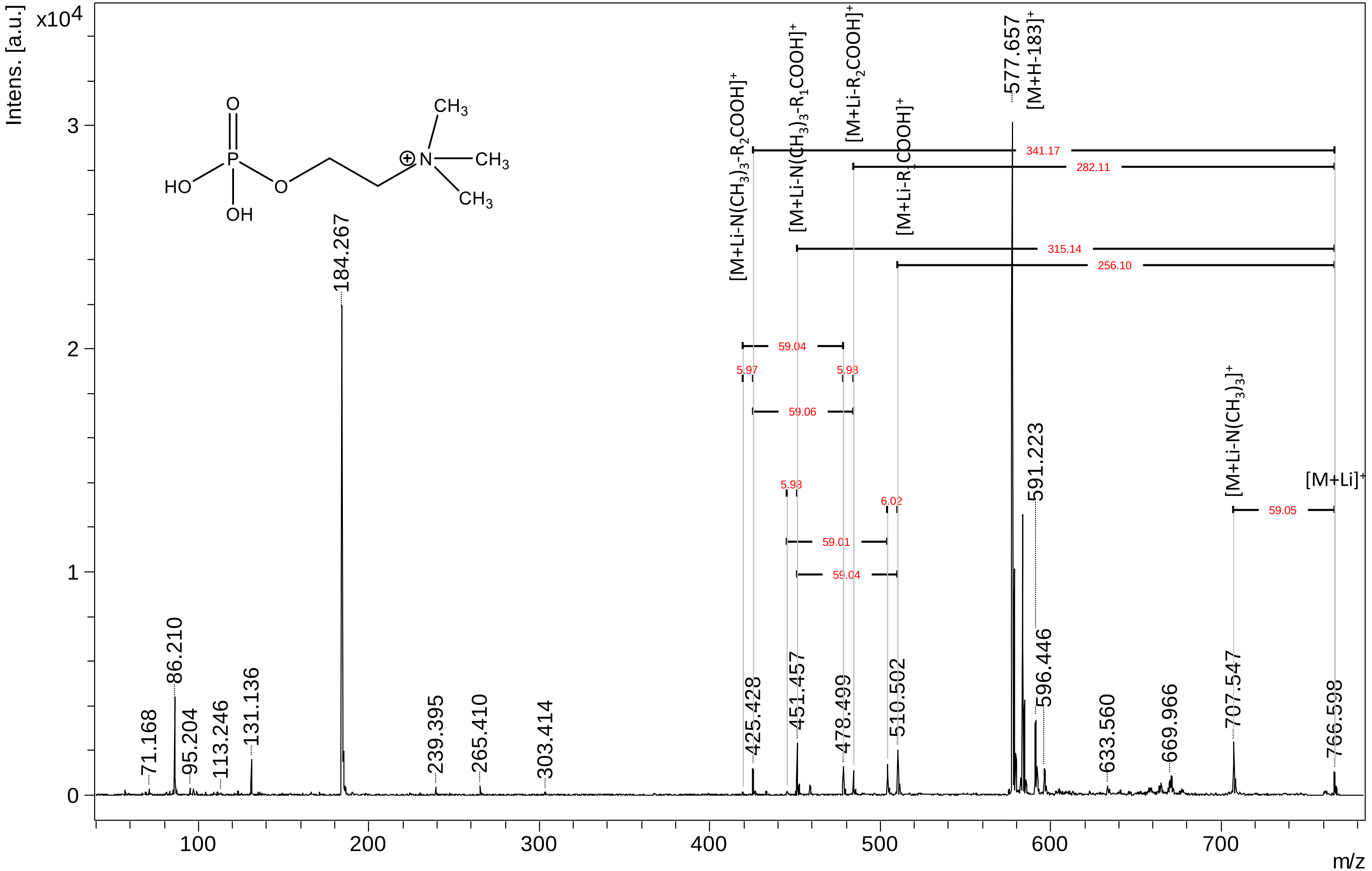

PC [16:0/18:1]
$[M+K]^+$ 798

Theoretical Mass: 798.53954 ($C_{42}H_{82}NO_8PK$)
Observed Mass: 798.5410
Δppm = 1.8

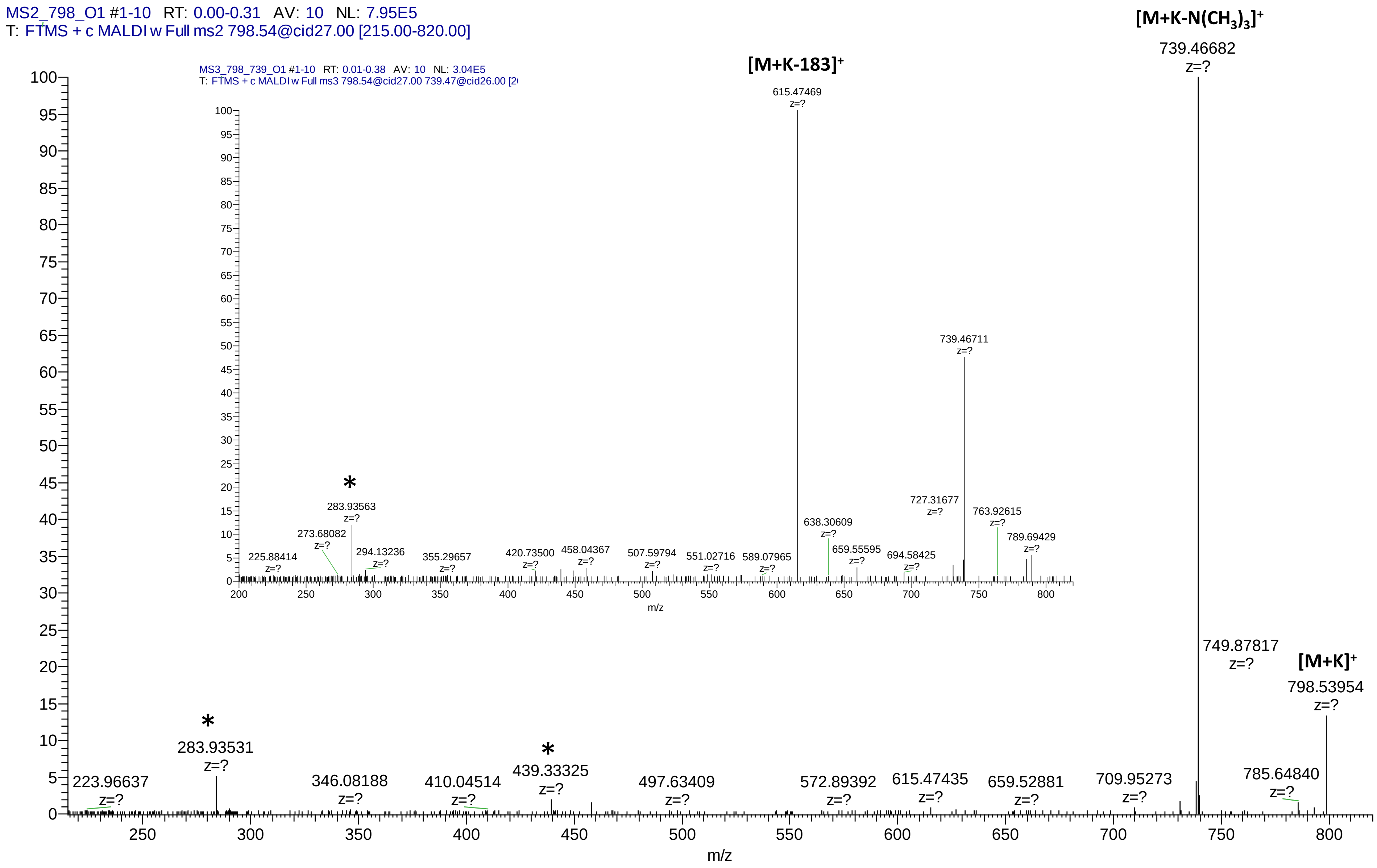

d18:1/24:0 - GalCer White Matter-specific ceramide
$[M+H]^+$ 812, $[M+Li]^+$ 818

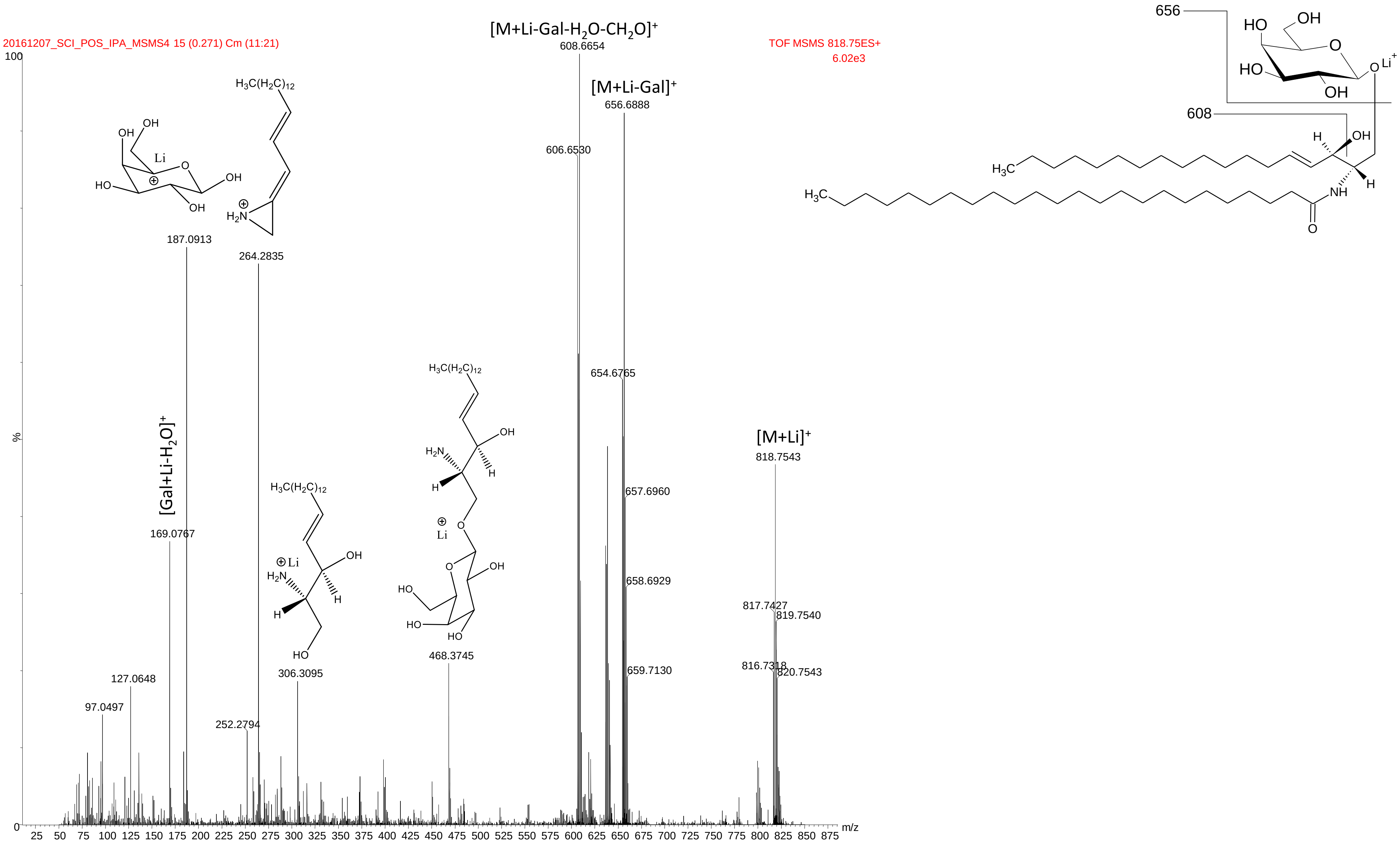

Heme-containing fragment
[M+H]$^+$ 616

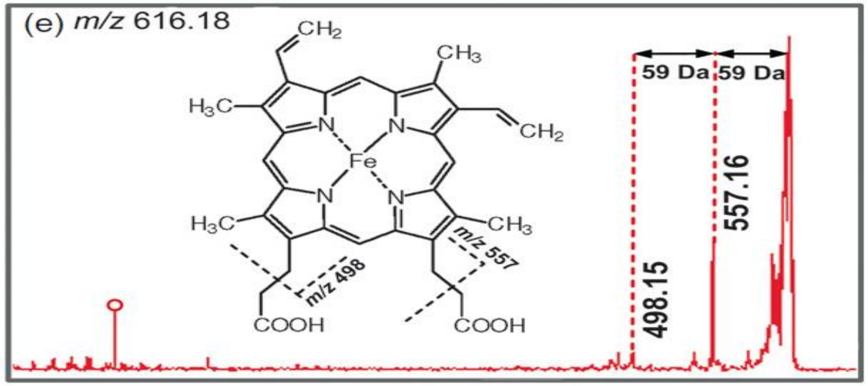

Ye Li et al 2013

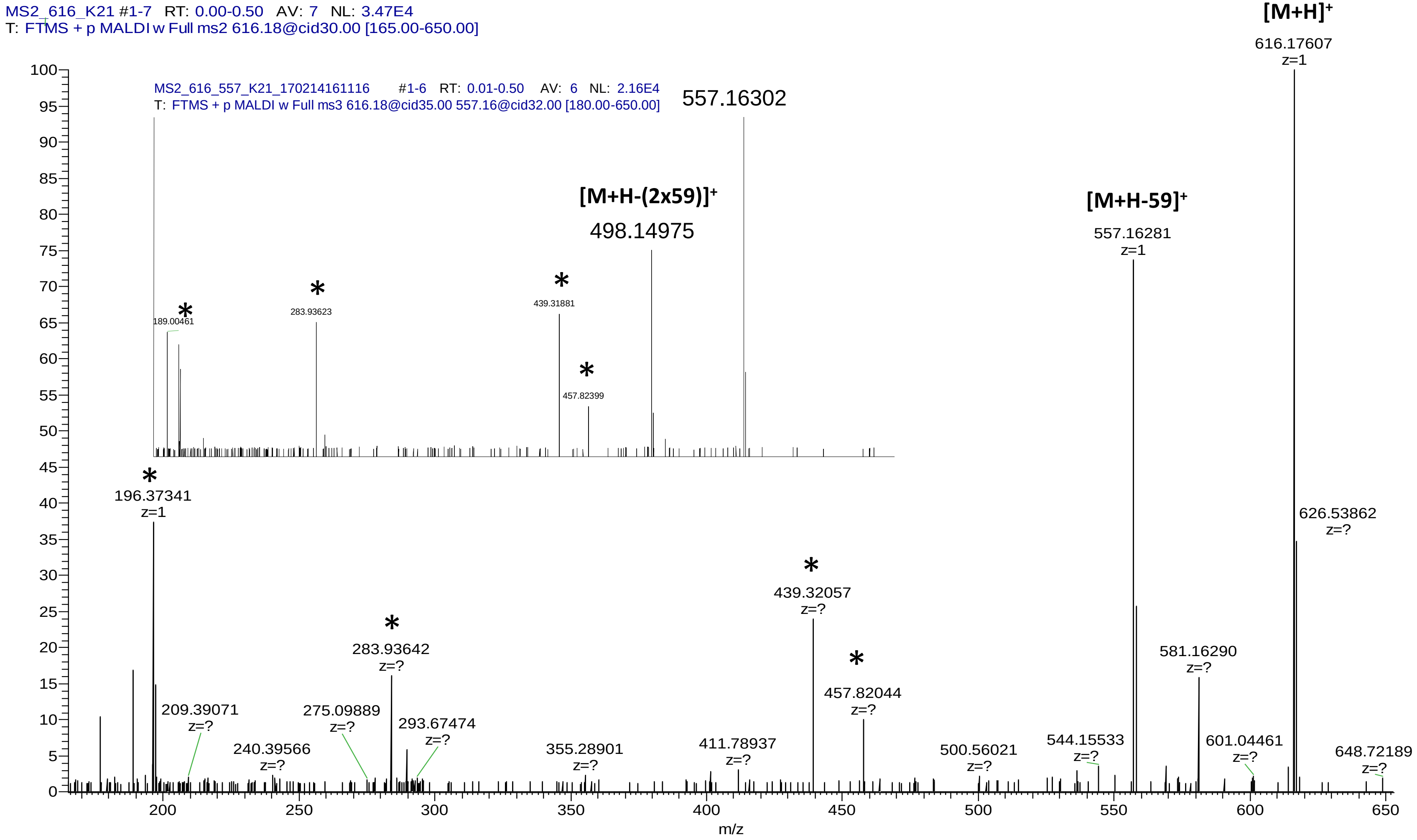

PC [38:4]
[M+Na]+ 768

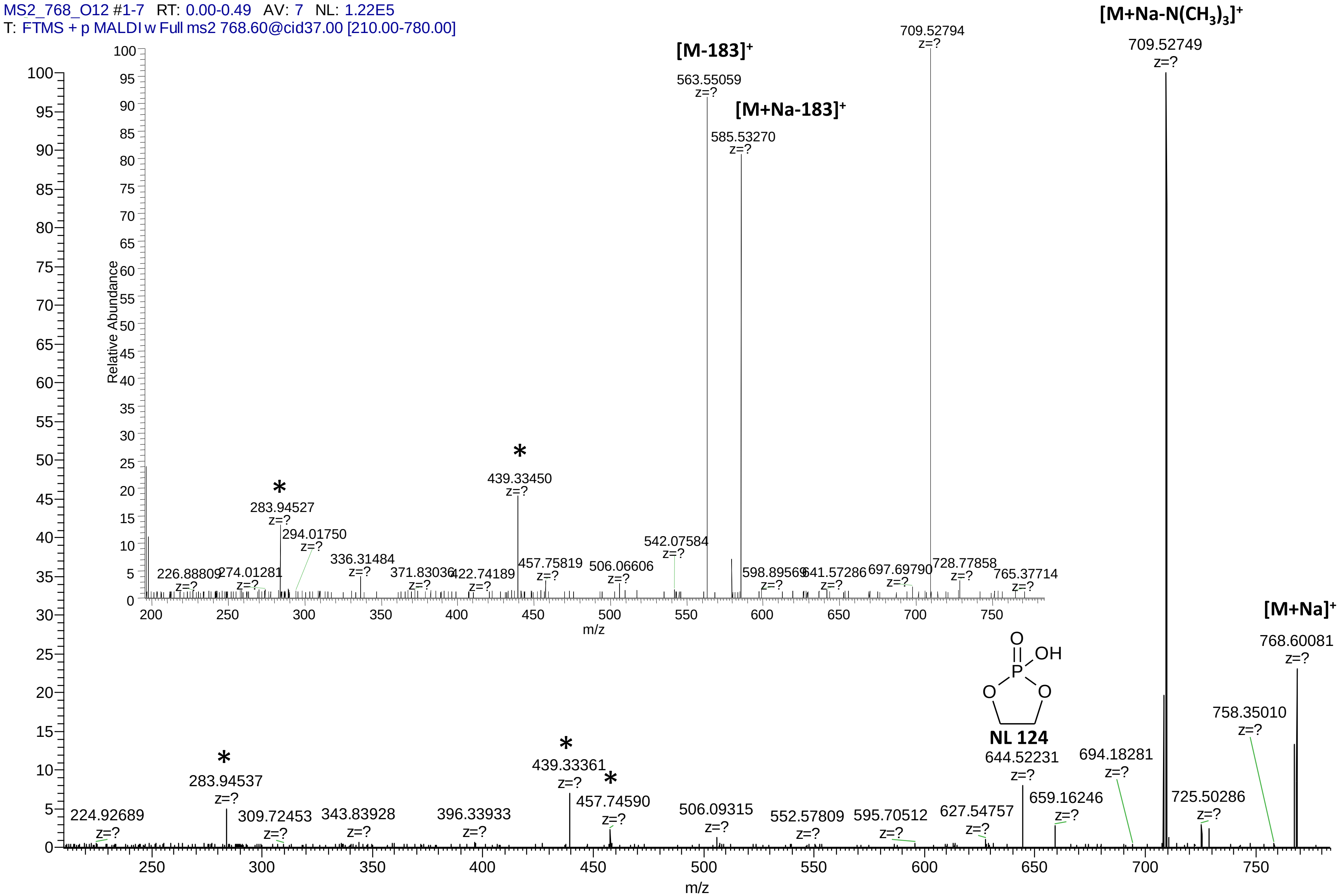

Supplement: Supplementary file 1 — Supplementary Information [file 41598_2018_34518_MOESM1_ESM.pdf]
